# Putative trehalose biosynthesis proteins function as differential floridoside-6-phosphate synthases to participate in the abiotic stress response in the red alga *Pyropia haitanensis*

**DOI:** 10.1186/s12870-019-1928-2

**Published:** 2019-07-19

**Authors:** Minxiu Sun, Zhujun Zhu, Juanjuan Chen, Rui Yang, Qijun Luo, Wei Wu, Xiaojun Yan, Haimin Chen

**Affiliations:** 1 Key Laboratory of Marine Biotechnology of Zhejiang Province, Ningbo University, Post Box 71, Ningbo, 315211 Zhejiang Province China; 2 Ningbo Institute of Oceanography, Ningbo, 315832 Zhejiang China

**Keywords:** *Pyropia haitanensis*, (Iso)floridoside, Trehalose-6-phosphate synthase, Floridoside-6-phosphate synthase, Abiotic stress

## Abstract

**Background:**

The heteroside floridoside is a primary photosynthetic product that is known to contribute to osmotic acclimation in almost all orders of Rhodophyta. However, the encoding genes and enzymes responsible for the synthesis of floridoside and its isomeric form, l- or d-isofloridoside, are poorly studied.

**Results:**

Here, four putative trehalose-6-phosphate synthase (TPS) genes, designated as *PhTPS1*, *PhTPS2*, *PhTPS3,* and *PhTPS4*, were cloned and characterized from the red alga *Pyropia haitanensis* (Bangiophyceae). The deduced amino acid sequence is similar to the annotated TPS proteins of other organisms, especially the UDP-galactose substrate binding sites of PhTPS1, 2, which are highly conserved. Of these, PhTPS1, 4 are involved in the biosynthesis of floridoside and isofloridoside, with isofloridoside being the main product. PhTPS3 is an isofloridoside phosphate synthase, while PhTPS2 exhibits no activity. When challenged by desiccation, high temperature, and salt stress, PhTPS members were expressed to different degrees, but the responses to thermal stress and desiccation were stronger.

**Conclusions:**

Thus, in *P. haitanensis*, *PhTPS*s encode the enzymatical activity of floridoside and isofloridoside phosphate synthase and are crucial for the abiotic stress defense response.

**Electronic supplementary material:**

The online version of this article (10.1186/s12870-019-1928-2) contains supplementary material, which is available to authorized users.

## Background

Red algae, which are ancient and highly populous eukaryotes, are widely distributed in coastal and continental areas from the tropics to the poles [1]. During photosynthesis, red algae fix inorganic carbon via the common plant enzyme ribulose-bisphosphate-carboxylase/oxygenase. However, the subsequent carbon flow into low-molecular-weight carbohydrates is much more diverse compared to other algal groups [2]. Galactosyl glycerol (GalG) is a low-molecular-weight carbohydrate that is the primary and most common soluble photosynthetic molecule in red algae. It is prevalent in the majority of Rhodophyta; for example, Cyanidiophyceae and Porphyridiophyceae only accumulate GalG [3], and its content in marine algae usually ranges from 1.5 to 8% on a dry-weight basis.

GalG has three different structures, including floridoside [α-d-galactopyranosyl-(1, 2)-glycerol], d-isofloridoside, and l-isofloridoside. The biosynthesis of GalG has long interested researchers. Using classical radioisotopes, previous studies have shown that exogenous inorganic ^14^C can be rapidly taken up and assimilated into floridoside [4], demonstrating that there must be enzymes that synthesize it. Marin et al. (1998) reported glucosyl-glycerol-phosphate synthase (GGPS) genes synthesizing similar compound-glucosyl-glycerol-phosphates in the cyanobacterium *Synechocystis* sp. [5]. The floridoside phosphate synthase (FPS) genes catalyzing the synthesis of (iso) floridosides were first reported by Pade et al. [6] They found two genes (*Gasu_26940* and *Gasu_10960*) in the red alga *Galdieria sulphuraria* that were annotated as trehalose 6-phosphate synthase (TPS)-like enzymes, but functioned as floridoside and isofloridoside phosphate synthase. However, among macroalgae, it remains unclear whether (iso)floridoside is synthesized by the same enzymes and pathway as in the unicellular *G. sulphuraria*. It is believed that floridoside biosynthesis involves the transfer of a galactosyl-unit from UDP-Gal to glycerol-3-phosphate (G3P). It is well known that isofloridoside has d- and l-isomeric forms and should thus be determined by the configuration of glycerol-3-phosphate [7]. The biosynthesis of floridoside and l-isofloridoside is initiated by a condensation reaction of l-glycerol-3-P and UDP-galactose, resulting in floridoside-P (sn-2) and l-isofloridoside-P (sn-1), respectively. These are subsequently de-phosphorylated by specific phosphatases. In both anabolic pathways, l-glycerol-3-P serves as a precursor, while d-glycerol-3-P should be the only source of sn-l glycerol-P (d-glycerol) in intermediary metabolism for the biosynthesis of d-isofloridoside. It is thus uncertain whether two floridoside phosphate synthases are sufficient to yield three structures of GalG-P or if more enzymes or multiple enzyme functions are required.

Previous research revealed that the biosynthesis of trehalose and (iso)floridoside involves similar substrates and reaction mechanisms. The two genes (*Gasu_26940* and *Gasu_10960*) discovered by Pade et al. [6] were initially annotated as TPS in the genome of *G. sulphuraria*. Using BlastP searches, several genes were also annotated as TPS in other red algae. For example, two putative TPS genes were screened out from the library of *Pyropia yezoensis* and *Saccharina japonica* (SjaTPS) by Deng et al. [8, 9] and were even cloned in vitro*.* It was reported that *Rhizoma salviae* possesses key enzymes for synthesizing low-molecular-weight sugars, but functional studies were not able to verify if the enzyme with the gene annotated as TPS has the ability to synthesize trehalose [10]. Hence, due to the lack of information on FPS genes, it is likely that the annotation of many genes remains inaccurate at present, and it is thus necessary to explore more FPS genes by functionally verifying the genes annotated as TPS in red algae.

The function of floridoside is similar to that of sucrose in higher plants. It is a stable and low-molecular-weight intermediate that serves as a dynamic carbon pool used by the cells as a carbon precursor in the biosynthesis of starch and cell wall polysaccharides [11]. Additionally, floridoside is accumulated at high amounts under stress conditions, such as high salinity, desiccation, and high temperature, and also functions in adjusting osmotic pressure, which is similar to trehalose in plants [12]. However, floridoside and isofloridoside have different functions. It was reported that floridoside acts as an osmoregulator in most red algae. Within Bangiales, floridoside is metabolically much more active than isofloridoside. Studies on the effects of salinity on the concentration of heterosides in Bangiales show that only floridoside plays an important role in osmotic acclimation, whereas the amount of isofloridoside remains almost unchanged [13, 14]. Moreover, heteroside patterns in red algae vary according to the differences in biogeographic regions, species and seasons. These findings suggest that the enzyme activities for catalyzing the biosynthesis of (iso)floridoside or the expression of genes responsible for the enzymes may differ under different stresses, species, or even seasons. In order to elucidate the reason for the diversity of floridoside molecules in red algae, genes encoding the floridoside biosynthetic enzymes, their expression profiles, and the activities of these enzymes should be analyzed.

In previous research on *Pyropia haitanensis*, and we found that the contents of floridoside and isofloridoside varied markedly under desiccation and high temperature stress [15]. However, the genes encoding (iso)floridoside biosynthetic enzymes remain unknown. Therefore, in this study, we retrieved four unigenes annotated as putative TPS-related genes from the transcriptome data of *P. haitanensis*. We attempted to identify these genes and evaluated their activity under different stresses to reveal their functions.

## Results

### Protein sequences and alignments

**Fig. 1:**
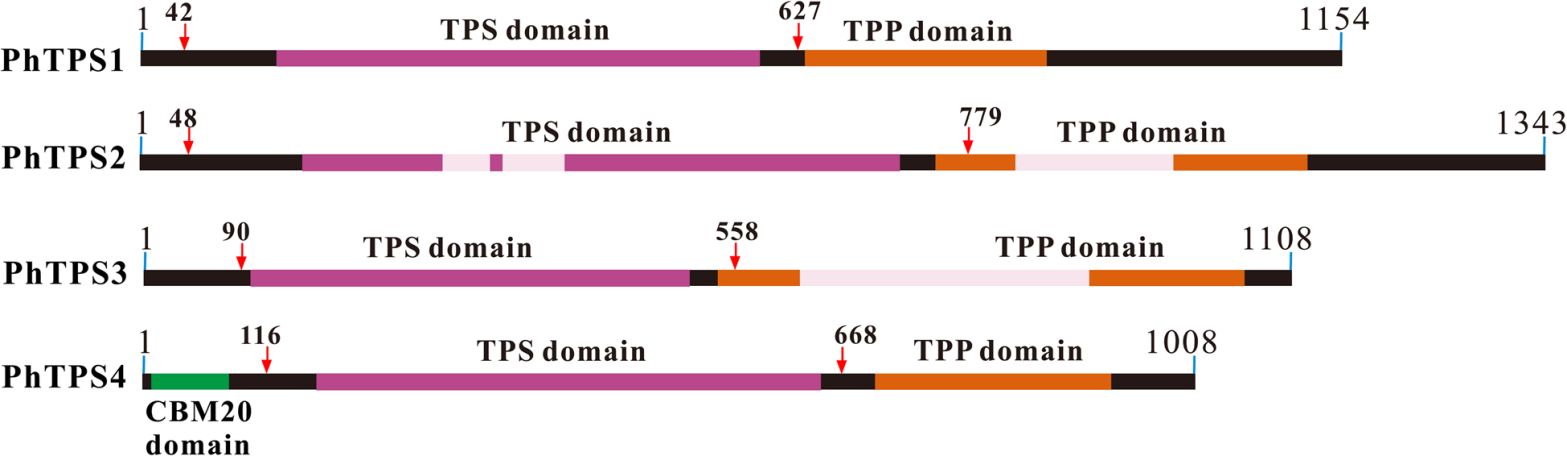
Conserved domains in the *P. haitanensis* TPS family. The black bar indicates the amino acid sequence length of the complete ORF of PhTPS1*–*4; the red arrows on the bar indicate the recombinant expression region; the green, purple, and orange area on the bar show the CBM20, TPS, and TPP functional domains, respectively; the light pink area in the domains indicates that the related sequence segment had low homology with the conserved superfamily recorded in NCBI

Four putative trehalose-6-phosphate synthase genes from *P. haitanensis* were cloned and named as *PhTPS1, PhTPS2, PhTPS3,* and *PhTPS4*. Their GenBank accession no. are KF147832.1, KM519457.1, KM519458.1, and KF245464.1, respectively. The open reading frames (ORFs) of *PhTPS1–4* are 3462 bp, 4029 bp, 3324 bp, and 3024 bp in length and encode polypeptides of 1154 aa, 1343 aa,1108 aa, and 1008 aa, respectively (Fig. 1). The molecular weights of the PhTPS1*–*4 deduced amino acid sequences are 124, 145, 117, and 112 kDa, with a theoretical isoelectric point (pI) of 6.73, 6.05, 5.99, and 5.77, respectively.

By searching in the NCBI Conserved Domain Search tool, two conserved structural domains named TPS domain (Glyco_transf_20) and TPP domain (Trehalose_PPase) were discovered in PhTPS1, PhTPS2, and PhTPS3 (Fig. 1). The TPS domain in the three PhTPS comprises the main length of the protein and is present near the N-terminal and is annotated as trehalose-6-phosphate synthase. The TPP domain annotated as trehalose-6-phosphate phosphatase (TPP domain) is located at the C-terminal, whereas in PhTPS4, with the exception of the two domains, there is a special domain named CBM20 at the N-terminal known to be involved in starch binding.

Currently, only the crystal structures of *Escherichia coli* TPS (PDI No. 1GZ5) [16] and *Candida albicans* TPS (PDI No. 5HUT) [17] proteins have been elucidated. Here, we compared the data of these two proteins and used multiple sequence alignment to evaluate the TPS domains from different species and GGPS domain sequences from cyanobacteria. It was found that the TPS domains of PhTPS1–3 were homologous with the *C. albicans* TPS (PDI No. 5HUT) protein, with 53, 50, and 34% identity, respectively. However, PhTPS4 showed a low identity of only 10%. Based on the alignment, we found nine sites conserved with the UDP-glucose substrate binding sites, and four sites conserved with the glucose-6-phosphate binding sites (Additional file 1: Figure S1). Sites G157, D274, H298, R406, D505, M507, N508, L509, and E513 of PhTPS1, and sites G181, D410, H434, R542, D641, M643, N644, L645, and E649 of PhTPS2 were associated with the substrate UDP-glucose binding sites and are highly conserved, without any mutated sites. However, in the two proteins PhTPS3 and PhTPS4, there are three different sites. For example, in PhTPS3, D201, H225, and N441 are changed to N, Y, and S, respectively. In PhTPS4, the mutated sites are G198K, R464D, and M564 L, but the other six sites (D328, H352, D562, N565, L566, E570) are conserved. For the substrate glucose-6-phosphate binding sites, only four sites in PhTPS1 (R136, Y213, W222, and R440) are highly conserved. In PhTPS2–4, site mutations were present, including Y247H and R580Q in PhTPS2, R63H and Y141F in PhTPS3, while R501 in PhTPS4 is deleted. In addition to the binding sites of the two substrates, multiple sequence alignment showed that the sequences of the four PhTPS members were highly similar to the highly conserved fragments (homology > 90%) of other species TPSs and cyanobacteria GGPSs. An insert fragment (309–397 aa) was found in PhTPS2. This insert was also found in *P. yezoensis* TPS-3 (contig_27879) (350–427 aa, with 48% identity with the PhTPS2 insertion fragment). No other species were detected.

### Phylogenetic analysis of trehalose-6-phosphate synthase in *P. haitanensis*

**Fig. 2:**
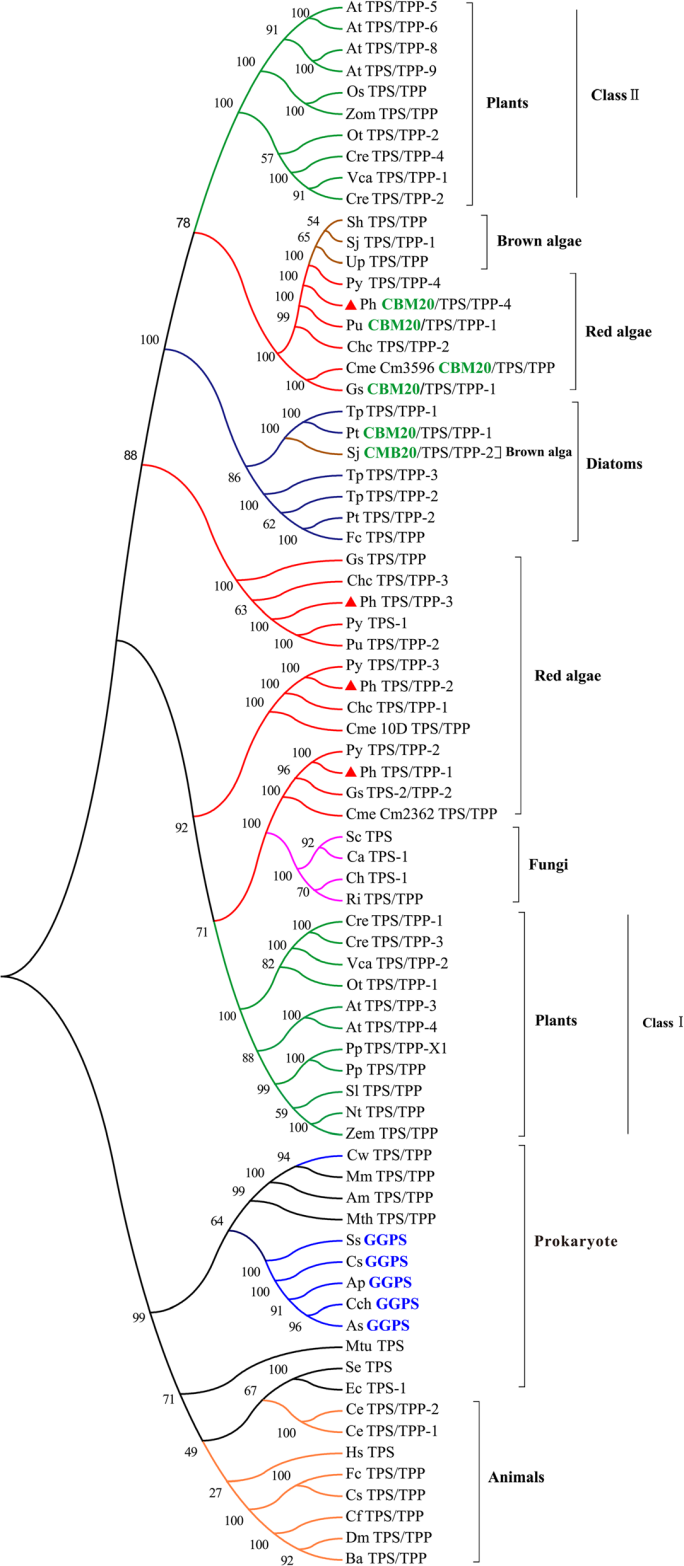
Phylogenetic analysis and structural evolution of TPS、TPS/TPP and GGPS proteins from different species. A NJ tree was constructed to show the phylogenetic relationships of the TPS、TPS/TPP and GGPS proteins using the functional-related full amino acid sequences from prokaryotes, red algae, diatoms, brown algae, fungi, green algae, plants, and animals. Their accession numbers are indicated in Additional file 3: Table S1. There were 1,000 bootstrap replicates. The red triangle shows PhTPS1*–*4. The functional domain in each sequence was retrieved using the Conserved Domain tool in NCBI and is marked by a superscript

In this study, a phylogenetic tree of the fused protein from bacteria, fungi, algal, animals, and higher plants was constructed based on the TPS/TPP, TPS, and GGPS domain to investigate the evolutionary relationships among them (Fig. 2). Single domain TPS proteins were mainly located in the group of prokaryotic sequences. TPS/TPP fused proteins existed extensively.

The tree is separated into two main clades. The TPSs of animals and some prokaryotes and the GGPSs of the cyanobacteria form one clade. In this clade, the special GGPSs are grouped at the end of a single branch. The TPSs of some prokaryotes, fungi, algae, and higher plants form another cluster. Prokaryotic fused TPS/TPP proteins are located between the single domain prokaryotic sequences and all of the eukaryotic sequences. The TPS genes of plants are divided into two very distant clades that belong to plant Class I enzymes and plant Class II enzymes. It is clear that the proteins from red algae are closely related to each other, but group in clusters; for example, *P. yezoensis*, *Chondrus crispus*, *G. sulphuraria,* and C*yanidioschyzon merolae*. PhTPS1 and 2 are close to the proteins of *P. yezoensis* (Contig 4636 and Contig 27879) with homologies of 81.28 and 72.3%. Four clusters, namely, PhTPS1–4 are dispersed along different branches instead of clustering together. The cluster including PhTPS1–2 is along the branch with plant Class I, and PhTPS3–4 is along the branch with plant Class II. PhTPS4 is relatively distant from the other three PhTPSs and is closely associated with Class II proteins. It forms a small cluster with some red and brown algae. Some TPS proteins containing the N-terminus CBM20 domain were noted. They are relatively close in the phylogenetic tree, involving proteins from red algae, diatoms, and brown alga (*S. japonica*); for example, *P. umbilicalis* (OSX79290.1, 85.84%), *G. sulphuraria* (EME31717.1, 48.05%), and C*. merolae* CM3596 (BAM80147.1, 41.25%) from Rhodophyta, *S. japonica* (AGT20052.1, 23.45%) from Phaeophyta, and *Phaeodactylum tricornutum* CCAP 1055/1 (XP_002180425.1, 28.36%) from Bacillariophyta, but are not found in the TPS genes of other species.

The phylogenetic tree of the only TPS and TPP domain were also constructed, respectively (Additional file 2: Figure S2A, B). It could be found that the phylogenetic tree for only TPS domain is nearly the same as that of TPS/TPP. While, the phylogenetic tree for only TPP domain is different from that of TPS/TPP. Instead of forming two large clades, all clades branched from the root and the clades position changed. For example, the PhTPP domains are divided into three clades. The clades of PhTPP 1 and PhTPP 3 are separated by plant Class I. Among them, the cluster of red alga including PhTPP 1and plant Class I to form a clade. Besides, the species of each small clade is basically the same.

### Expression and enzymatic function of PhTPS1–4 proteins

**Fig. 3:**
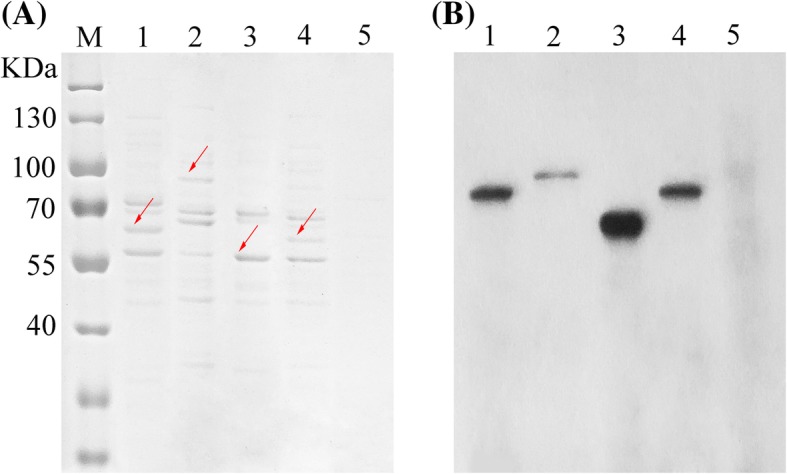
Analysis of the reaction products of the in vitro translation of the TPS domain from PhTPS1*–*4 of *P. haitanensis* using the *E. coli* BL21 cells expression system by SDS-PAGE. **a** Coomassie-stained gel. The arrows indicate proteins of the expected size for *PhTPS1–4* (lane M, Maker; lane 1–4, PhTPS1–4, lane 5, negative control, with empty pET-28a or pET-28a-sumo vector). **b** Translated proteins were analyzed by western blot using a His-tag-specific antibody

To verify the function of four proteins from *P. haitanensis* PhTPS1–4, we expressed their TPS domain by *E. coli* and separated the purified proteins by SDS-PAGE. We observed bands in the position of the corresponding molecular weight (PhTPS1, 77.9 kDa; PhTPS2, 82.3 kDa; PhTPS3, 65.4 kDa; PhTPS4, 75.9 kDa). To confirm the expression, four recombinant His-tagged proteins were confirmed by Western blotting using an anti-His-tag-antibody (Fig. 3).

**Fig. 4:**
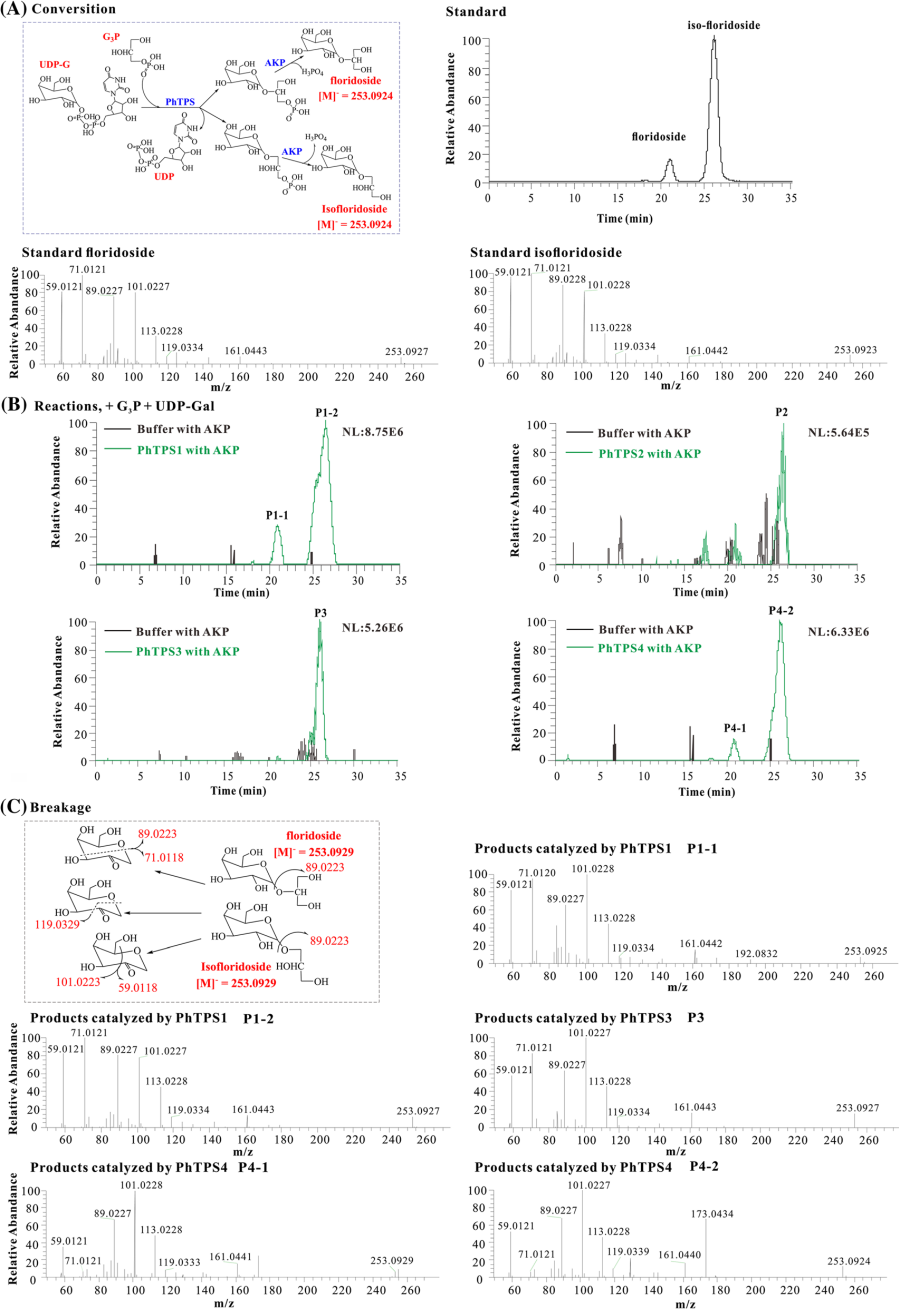
LC–MS analysis of the products catalyzed by PhTPS1*–*4. **a** The synthetic pathway of (iso)floridoside, total ionization chromatogram (TIC), and MS/MS spectra of floridoside and isofloridoside standards. **b** TIC of (iso)floridoside produced by PhTPS1–4 catalyzation. **c** The breakage of (iso)floridoside and the PhTPS1, 3, 4-catalyzing MS/MS spectra of (iso)floridoside

To detect the biochemical activity of PhTPS1–4, UDP-Gal and G3P were allowed to react with them, and the resulting products were respectively analyzed (Fig. 4). First, the retention times of the two purified standards, floridoside (retention time = 20.83 min) and isofloridoside (retention time = 26.14 min), were obtained and identified using the [M-H]^−^ ions at *m/z* 253.0925 by HPLC-MS. In MS/MS spectra, the characteristic fragment ion at *m/z* 89.02 and 119.03 from [M-H]^−^ ions were also utilized for qualitative analysis of floridoside and isofloridoside [18]. It was found that the reaction products floridoside and isofloridoside were generated which were catalyzed by PhTPS1 and PhTPS4 using HPLC-MS. While catalyzing by PhTPS3, only the isofloridoside was produced. However, the floridoside and isofloridoside were not detected after catalyzing by PhTPS2.

Quantitative analysis of the catalytic products of PhTPS1, PhTPS3, and PhTPS4 showed that the conversion ratios of the four enzymes were all low. The enzyme activities of PhTPS1 and PhTPS4 producing floridoside were 0.26 and 0.22 μmol·h^− 1^·mg^− 1^, respectively. The enzyme activities of PhTPS1 and PhTPS4 producing isofloridoside were 0.50 and 0.61 μmol·h^− 1^·mg^− 1^, respectively. The enzyme activity of PhTPS3 was 0.23 μmol·h^− 1^·mg^− 1^, and only the isofloridoside was biosynthesized (Table 1).

**Table 1:** Quantitative determination of PhTPS 1–4 catalytic products

Enzyme	Product (μM)		Enzyme activity (μmol·h^−1^·mg^−1^)	
	Floridoside	Isofloridoside	Floridoside	Isofloridoside
PhTPS1	142.8 ± 18.5	275.1 ± 17.2	0.26 ± 0.02	0.50 ± 0.02
PhTPS2	ND	ND	ND	ND
PhTPS3	ND	196.0 ± 23.1	ND	0.23 ± 0.03
PhTPS4	119.6 ± 16.1	272.3 ± 14.2	0.22 ± 0.02	0.61 ± 0.01

*ND* None detected

### Expression of *PhTPS1*–*4* under different abiotic stimuli

**Fig. 5:**
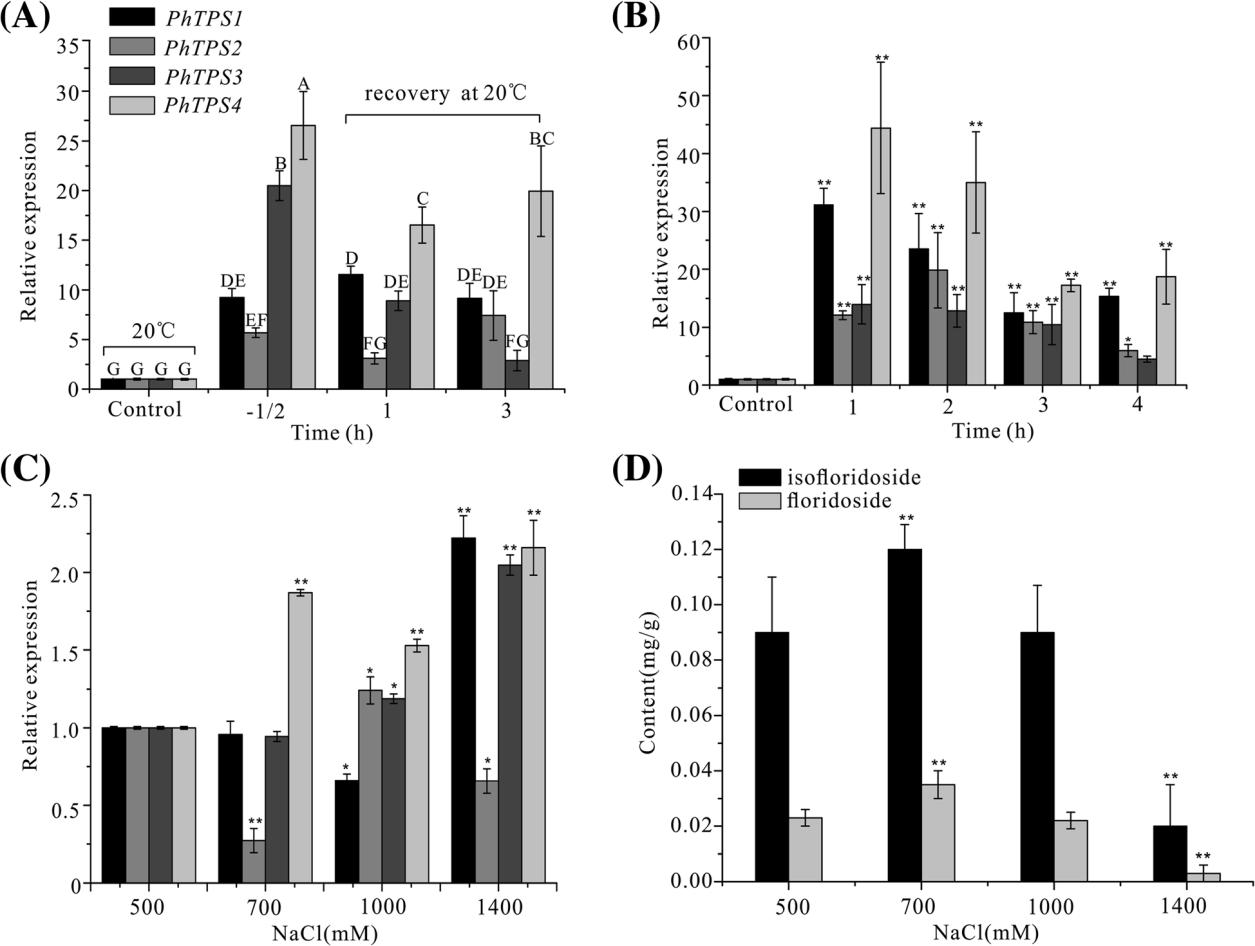
Effects of different abiotic stresses on the expression of PhTPS1–4 and content of (iso)floridoside. **a** Relative expression of PhTPS1–4 under high temperature stress. The thalli of *P. haitanensis* were treated at 35 °C for 30 min and subsequently allowed to recover at 20 °C for 1 and 3 h. Control, *P. haitanensis* cultured in sterilized seawater at 20 °C; − 1/2, Samples exposed to 35 °C for 30 min; 1 and 3 h for recovery time. Values denoted with different letters indicate significant differences according to Tukey’s multiple comparison tests. **b** Relative expression of PhTPS1–4 under desiccation. The thalli of *P. haitanensis* were subjected to desiccation for 0, 1, 2, 3, and 4 h under 20 °C, 100 μmol photons m^− 2^ s^− 1^, and 75% humidity. **c** Relative expression of PhTPS1–4 under 500 to 1400 mM salt stress for 1 h under 20 °C. **d** Effect of salt stress on the production of (iso)floridoside in *P. haitanensis.* The thalli of *P. haitanensis* were cultured in medium supplemented with 500 to 1400 mM NaCl for 1 h under 20 °C. The bars in (**b**, **c**, and **d**) represent the mean of three biological replicates and three technical repetitions (*n* = 3), and the error bars depict one SD. One-way ANOVA, *P < 0.05, **P < 0.01 (relative to the control)

The expression of four *PhTPS* genes was analyzed under desiccation, high temperature, and different salinity treatments (Fig. 5). Following 35 °C high temperature stress treatment for 30 min, the expression of *PhTPS1–4* was significantly increased. The increase in *PhTPS3* and *PhTPS4* was the most obvious, reaching 20.5- and 26.6-fold (*P* < 0.01) that of the control, followed by *PhTPS1*, which was increased by 9.2-fold (*P* < 0.01) that of the control. After recovery under normal temperature for 1 h following the thermal shock, the upregulation of *PhTPS2, PhTPS3,* and *PhTPS4* was reduced, but the upregulation of *PhTPS1* was significantly enhanced, reaching 11.5-fold that of the control. Compared with recovery for 1 h, recovery for 3 h did not elicit any major changes (Fig. 5a).

During the first 1 h of desiccation, the expression of four *PhTPSs* increased significantly and remained at high levels throughout the process. Among them, *PhTPS1* and *PhTPS4* showed the strongest responses. When treated for 1 h, the increased expression multiple reached more than 30 times (*P* < 0.01), and the expression level gradually decreased with the extension of desiccation time. However, the up-regulation of *PhTPS2* and *PhTPS3* was slightly weaker than that of *PhTPS1* and *PhTPS4*, and the up-regulation peaked at 2 h, but the up-regulation remained at around 5–12 fold of the control during the entire desiccation process (Fig. 5b).

The expression of the PhTPS1–4 genes was examined when the *P. haitanensis* thalli were grown under different NaCl concentrations ranging from 500 mM to 1400 mM (Fig. 5c). *Pyropia haitanensis* is mainly grown in the East China sea, and the seaweed used in this study is from Xiangshan, China, where the salinity is 500 mM. Therefore, here we compare gene expression under different salinity stress concentrations with that under 500 mM NaCl as a control. According to the results, the expressions of four *PhTPS* genes varied under different salinity stresses, but their overall expression was not very high. Among them, *PhTPS4* was most sensitive to changes in salinity, and under 700 mM NaCl, *PhTPS4* showed slight salt-stimulated expression and was upregulated to 1.86-fold that of the 500 mM NaCl group (*P* < 0.01). The levels of *PhTPS1*, *PhTPS3,* and *PhTPS4* were increased under 1400 mM NaCl stress, being 2.22-, 2.04-, and 2.16-fold higher than that of the 500 mM NaCl group (*P <* 0.01). *PhTPS3* and *PhTPS4* all reacted relatively strongly at high salinity. *PhTPS2* was not upregulated with the increase in salinity in comparison to the 500 mM NaCl group.

The accumulation of (iso)floridoside in *P. haitanensis* under various NaCl concentrations ranged from 500 mM to 1400 mM for 1 h. LC–MS revealed that floridoside and isofloridoside all accumulated (Fig. 5d). The concentration of isofloridoside rose proportionally with the external NaCl from 500 mM to 700 mM (*P* < 0.01), but decreased under highly hypersaline conditions. Floridoside plays a rather minor role as an osmolyte, because its change trend was the same as isofloridoside under salt stress and even decreased at a high salt concentration.

## Discussion

Studies have found that the biosynthesis of glucosylglycerol, trehalose, and (iso)floridoside has many similarities, including the selection of the substrate type, the catalytic enzymes, and the product structure [19]. Genetic evolution research also suggests that GGPS, TPS, and FPS genes in red algae, bacteria, and other eukaryotes have some degree of conservation (Additional file 1: Figure S1). For example, in the cyanobacteria *Synechocystis* sp. PCC 6803, there is a substance similar to the glycoside structure of the red algae, glucosyl-glycerol, and its synthetic gene has been identified [5]. Using GGPS to search for similar genes in red algal organisms, TPS genes were predominantly retrieved. Interestingly, although highly conserved genetic information with plant TPSs can be found in many red algae, the presence of trehalose is generally not detected in most red algae, especially in Bangiophyceae, and thus we speculated that the TPS genes in *P. haitanensis* may perform the function of synthesizing iso(floridoside). Barbier et al. compared the genomes of *G. sulphuraria* and *C. merolae*, two unicellular red algae, and speculated that TPS-like genes in red algae might be involved in the synthesis of floridoside [12]. Pade et al. [6] reported that two TPS-like genes, *Gasu_10960* and *Gasu_26940,* from the red alga *G. sulphuraria* encode the enzymatically-active floridoside and isofloridoside phosphate synthase/phosphatase fusion proteins. Many putative TPS gene sequences in red algae were subsequently reclassified into the FPS class.

Initial searching in the *P. haitanensis* transcriptome data suggested that four genes had the potential to encode the enzymes catalyzing (iso)floridoside synthesis, and sequence alignment showed similarity with the GGPS and TPS proteins of other organisms. Alignment and homology analysis of the TPS proteins crystal structure data of *E. coli* (PDI No. 1GZ5) and *C. albicans* (PDI No. 5HUT) with PhTPS proteins elucidated the sites related to substrate binding. The UDP-glucose substrate binding sites were highly conserved, particularly PhTPS1 and PhTPS2, and the nine binding sites were all matched. However, the sites involved in the binding with glucose-6-phosphate possessed relatively little conservation, as floridoside synthetic substrates include UDP-galactose and glycerol-3-phosphate. This result indicates that, during their evolution, the sites binding to UDP-glycoside in the enzymes for the synthesis of low-molecular-weight carbohydrates are relatively conservative, even if UDP-glucose is replaced by UDP-galactose. As there are structural differences between glycerol-3-phosphate and glucose-6-phosphate, several genes involved in the active center amino acids of enzymes may have mutated in order to fit the binding with the substrate.

Compared with the three phylogenetic trees including TPS/TPP, only TPS and TPP domains, it is evident that the evolution of four PhTPSs was not consistent. TPS domain is nearly the same as that of TPS/TPP fusion protein, indicating that the evolution of fused proteins is mainly affected by the TPS domain. The species of TPS and TPP in each small clade showed similar evolution, but the differences between clades meant that the evolution of TPP was not accompanied by TPS. For example, the evolution of PhTPP1 in red algae is more close to that of Class I in plants. In the fused proteins phylogenetic tree, especially PhTPS4, which is on a distant branch and contains a unique CBM20 domain. In Rhodophyta, floridoside is a transient product of carbon fixation in photosynthesis. This carbohydrate storage acts as a dynamic carbon pool for starch or cell-wall polysaccharide biosynthesis. However, in some cases, the carbon flux will return from the polysaccharide to floridoside [4, 20]. The isofloridoside phosphate synthase/phosphate of *P. haitanensis* (*PhTPS4*) and the closely related proteins of *C. merolae*, *G. sulphuraria*, *S. japonica*, and *P. tricornutum* all have a CBM20 domain at their N-terminus. CBM20s are linked to catalytic modules associated with starch or glycogen metabolism, such as being combined with alpha-amylase (EC 3.2.1.1) starch-degrading enzymes [21, 22]. Previous studies have also shown that in seaweed, the content of isofloridoside is significantly higher than that of floridoside [18]. This domain may be responsible for isofloridoside phosphate synthase/phosphatase in combination with floridean starch. This combination may contribute to the use of precursors [6]. During the degradation of floridean starch, UDP-glucose is produced, which is transformed into UDP-galactose by UDP-galactose-4-epimerase [12]. Interestingly, in the four PhTPS enzymes, only PhTPS4 containing the CBM20 domain catalyzes to produce just isofloridoside. Similarly, in the two genes in *G. sulphuraria*, only *Gasu_10960* containing the CBM domain catalyzed the production of isofloridoside, but no floridoside. This reveals that the precursors for the biosynthesis of isofloridoside may be partly derived from floridean starch.

The biosynthesis of (iso)floridoside is initiated by the condensation of G3P and UDP-galactose to the phosphorylated intermediate (iso)floridoside phosphate. This intermediate is subsequently dephosphorylated by a specific phosphatase. It was reported that floridoside or isofloridoside cannot be converted by intramolecular rearrangement [23, 24]. Moreover, isofloridoside has l- and d-configurations, which means that there should be different enzymes to catalyze their synthesis. Based on Blast searches, we found that there are several TPS genes in each red alga. Some of them may have lost their original functions during evolution, or some of them may perform different functions in alga. We found that the four PhTPS proteins in *P. haitanensis* did not show trehalose-6-phosphate synthase functionality. Wang et al. [10] also analyzed a TPS gene (PyTPS) and found that it also contained TPS/TPP domains, but appeared to lack TPS and TPP activity in yeast transformants. Similar to the findings of Pade et al. [6], PhTPS1, 3 and 4 all have floridoside phosphate synthase activities, but show certain differences. For example, PhTPS1 and PhTPS4 are able to catalyze the formation of floridoside and isofloridoside. However, isofloridoside was the main product catalyzed by the two enzymes, and isofloridoside catalyzed by PhTPS4 was 2.78 times higher than floridoside. PhTPS1can synthesize floridoside with a slightly higher content, whereas PhTPS3 only catalyzes the formation of isofloridoside. These results showed that the catalytic function of each enzyme exhibited favoritism and specificity, while PhTPS2 showed no FPS activity. Pade et al. [6] also found that, in the two TPS genes of *G. sulphuraria*, Gasu_10960 is consequently an isofloridoside phosphate synthase and Gasu_26940 is a floridoside and isofloridoside phosphate synthase. Chen et al. [18] detected floridoside and isofloridoside in *P. haitanensis*, and the content of isofloridoside was much higher than that of floridoside. In this study, we also found that the amount of isofloridoside catalyzed by PhTPS1, 4 was significantly higher than floridoside. However, because the catalytic efficiency of the four PhTPS proteins was very low, it was hard to distinguish and quantify the l- and d-isofloridoside catalyzed by the three PhTPSs, which remains to be further elucidated.

Recently, the evolutionary origin of trehalose biosynthesis genes was addressed. It was found that TPS/TPP fusion proteins exist widely in diverse organisms. Gene-fusion events have taken place at some point in the prokaryotic stage in the evolution of this family, which thereafter evolved to eukaryotes [25]. In plants, two subbranches are clearly distinguished; one group mainly includes plant Class I enzymes and the other includes Class II enzymes [25]. In contrast, algae show no clear evolutionary order, such as PhTPS1*–*2 being observed near Class I enzymes, whereas PhTPS4 was near Class II enzymes. PhTPS3 was in the middle of the two branches. One reason may be the absence of TPS or TPP catalytic activity in these enzymes, which lowered the evolutionary pressure. In plants, Class I and Class II proteins possess both TPS and TPP consensus regions, but Class I proteins harbor TPS-active enzymes with no significant TPP activity, while Class II proteins appear to have lost both TPS and TPP enzymatic activity during evolution [25]. In our case, the sequence of PhTPS1 with FPS activity was located near the plant Class I sequences branch. Curiously, no FPS enzymatic activity was indicated for the PhTPS2 protein. Although PhTPS4 has FPS enzymatic activity, it is located near the Class II proteins.

Apart from its role in organic carbon transport and/or as a short-term reservoir recycled to provide organic carbon for cellular needs, the accumulation of floridoside has been associated with improved stress tolerance toward abiotic stress [15]. As the major organic osmolyte in red algae, floridoside is used as an osmoprotectant. In most cases, high concentrations of floridoside accumulate following transferal from a hyposaline to hypersaline medium [26]. Floridoside and isofloridoside may function differently. For example, the closely related *Bangia atropurpurea* synthesized and accumulated high contents of floridoside under hypersaline conditions, while isofloridoside remained a minor compound [2]. Reed et al. revealed that although the relative concentration of isofloridoside was high in *Porphyra purpurea*, only floridoside responded to the changes in external salt concentration [27]. These findings indicate that within Bangiales, floridoside is metabolically much more active than isofloridoside. Pade et al. [6] processed *G. sulphuraria* with different NaCl concentrations and showed that *Gasu_10960* mRNA did not respond to changes in external salinity, while *Gasu_26940* mRNA exhibited slight salt-stimulated expression. They explained that the increase in floridoside could not be solely explained by the transcriptional stimulation of the synthesis enzyme. In the present study, we also found that the levels of *PhTPSs* increased only moderately at high salt concentrations, and the expression of *PhTPS2* remained almost unchanged. Our analysis of the contents of isofloridoside and floridoside showed that the response of floridoside was higher under salt stress. These results thus corresponded well to the view that floridoside, but not isofloridoside, is used as an osmoprotectant in red algae. However, the content of isofloridoside was much higher than floridoside in *P. haitanensis*. PhTPS 1, 3, and 4 mainly catalyze the synthesis of isofloridoside, and thus their down-regulated transcription under salt stress is reasonable.

Previously, we investigated the desiccation adaptation of *P. haitanensis* and found that floridoside was significantly accumulated during the process of dehydration [28]. In this research, the expression of four *PhTPS*s showed rapid and significant up-regulation, and maintained a high transcription level throughout desiccation. In particular, the levels of *PhTPS1* and *PhTPS4* were up-regulated most significantly, indicating that floridoside and isofloridoside may all be involved in the response to desiccation, with floridoside acting as an osmolyte, whereas the function of isofloridoside is unknown.

Marine algae are subjected to high temperature stress. Under this stress, the defense system is activated [15]. We previously found that the content of floridoside decreased slightly under high temperature, but increased significantly during the recovery process. Here, the transcriptional regulation of PhTPS members increased significantly when the thalli were heat shocked at 35 °C, but was reduced under recovery at 20 °C. This indicates that the carbon flow was fast when the thalli were subjected to high temperature stress, and thus floridoside inside the cells may be used to synthesize cell wall components first. The content of floridoside thus initially decreased, but gene expression was activated in response to promote the subsequent increase in floridoside accumulation at the recovery stage. This indicates that the regulation of *PhTPS* constitutes one important means of stress adaptation in a high temperature environment.

## Conclusions

We describe, for the first time, that annotated putative trehalose-6-phosphate synthase genes in red maroalgae can encode proteins to show specific (iso)floridoside-6-phosphate synthase activity, with the substrate binding sites being highly conserved. In *P. haitanensis*, three PhTPSs perform different activities to produce different floridoside structures. PhTPS1, 4 are (iso)floridoside phosphate synthases with isofloridoside as their main product. PhTPS3 is an isofloridoside phosphate synthase, and these corresponded well to the high content of isofloridoside in *P. haitanensis*. While PhTPS2 showed no activity. The different *PhTPS* expression modes under abiotic stress suggests that they may involved in the response to stresses in algae.

## Methods

### Materials

Experiments were performed with gametophytic *P. haitanensis* HML collected at Hepu, Xiangshan Harbor, Zhejiang Province, China (29°09′18″N, 121°54′05″W). Young fronds were collected, dried in the shade, and stored at − 20 °C. Before experiments, the thalli were rehydrated with sterile seawater and then healthy samples were cultivated at 20 °C for 24 h under 40 μmol photons m^− 2^ s^− 1^ with a 12 h:12 h (L:D) photoperiod.

### Total RNA isolation and cDNA synthesis

The total RNA was isolated from *P. haitanensis* HML gametophytes with RNAisoPlus Reagent (TaKaRa Bio Inc., Otsu, Japan) according to the manufacturer’s protocol. The cDNA for the full-length sequence cloning and transcriptional analysis was synthetized by using SMARTer™ rapid amplification of cDNA ends (RACE) cDNA Amplification Kit (Clontech Laboratories, Inc., Palo Alto, CA, USA) and TaKaRa PrimeScript RT reagent kit (TaKaRa, Tokyo, Japan) according to the instruction manual, respectively.

### Full-length cDNA cloning of *PhTPS*

Based on the gametophyte transcriptome analysis of *P. haitanensis* (data not shown), four fragment sequences were annotated as trehalose-6-phosphate synthase genes (*PhTPS1–4*). Gene-specific primers, indicated in Table 2, were designed to clone the complete open reading frame (ORF) of *PhTPS1–4* using the 5′- and 3′-RACE method (SMART RACE cDNA Amplification Kit, Clontech). All of the PCR products were then cloned into the pMD18-T vector (TaKaRa, Dalian, China) for sequencing (Sangon Biotech, Shanghai, China).

**Table 2:**
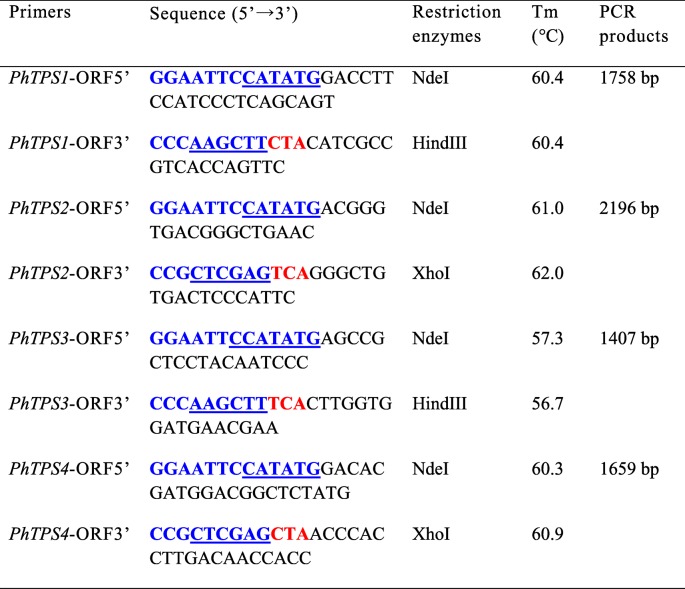
PCR primers for the amplification and cloning of *PhTPS1–4*

The underlined bases indicate the restriction sites, and the red bases indicate the added stop codons

### Analysis of *PhTPS* deduced amino acid sequences

The ORF in *PhTPS1–4* was analyzed using ORF Finder in the NCBI database. The theoretical molecular weights and pIs of the *PhTPS1–4* deduced amino acid sequences were calculated by the Compute pI/Mw tool at https://web.expasy.org/compute_pi/. The conserved structural domains were constructed in NCBI Conserved Domain Search.

### Multiple sequence alignment and phylogenetic tree construction

Gene sequences annotated as trehalose-6-phosphate synthase from bacteria, algae, fungi, plants, and animals were retrieved and collected from a search in the NCBI database. Gene sequences annotated as glucosyl glycerol-phosphate synthase from cyanobacteria were also collected. The ORFs of all TPS and GGPS gene sequences were obtained in the NCBI ORF Finder and translated into amino acid sequences using MEGA 5.1.0 software. The conserved domains of all TPS and GGPS amino acid sequences were analyzed in NCBI Conserved Domain Search.

Multiple sequence alignment of TPS and GGPS from different species was performed by Vector NTIAdvance 11.5.1 software with default parameters and then edited by GeneDoc software to show the function-related conserved sites in these sequences. Sequences of TPS, TPP, TPS/TPP and GGPS from different species were aligned using the ClustalW algorithm, and a phylogenetic tree was constructed using the neighbor-joining distance method with 1,000 bootstrap replicates. PRABI (https://geno3d-prabi.ibcp.fr/cgi-bin/geno3d_automat.pl?page=/GENO3D/geno3d_home.html) was used for PhTPS1–4 protein sequence homology alignment.

### Preparation of the recombinant TPS domain protein of PhTPS

The primers shown in Table 3 were used to clone the TPS domain of PhTPS1–4. The PCR procedure was 95 °C for 3 min; followed by 35 cycles of 95 °C for 30 s, *T*_m_ for 35 s, 72 °C for 2 min, and then 72 °C for 10 min. The amplicon was inserted into the commercial pET-28a (TaKaRa) vector or modified pET-28a-sumo vector, and then transformed into *E. coli* BL21. The expression of the target protein was induced in the presence of 0.1 mM isopropyl thio-*β*-galactoside at 20 °C for 16–24 h. The cells were then harvested, lysed, and centrifuged. The purification of the target protein in the supernatant was operated successively by using the 6 × His-Tagged Protein Purification Kit (Cwbio, Beijing, China) and the AKTAxpress™ system with a HiLoad™16/600 Superdex™ 200 pg column (GE-Healthcare, USA). The eluted protein solution (50 mM Tris/HCl, 200 mM NaCl, pH 8.0) was assessed by both 10% SDS-PAGE and Western blotting with the anti-His tag antibody (Sigma Aldrich). The *E. coli* with empty pET-28a or pET-28a-sumo vector was used as the negative control.

**Table 3:** The primers for qRT-PCR

Primers	Sequence (5′ → 3′)	Tm (°C)	PCR products
*PhTPS1*-Q5′	AGTTTCCGTTTGTGTGGGTG	58.0	132 bp
*PhTPS1*-Q3′	CCGTTGTAGTAGAGGTGGGC		
*PhTPS2*-Q5′	TGCTGGGGGTGGAAGGG	59.0	196 bp
*PhTPS2*-Q3′	GGGGAAGGGGGTGTGGAG		
*PhTPS3*-Q5′	CTGCCCACTCGTTTTCCA	57.0	142 bp
*PhTPS3*-Q3′	CCGGCTCAATTTCTTCCAG		
*PhTPS4*-Q5′	TGTATGATGGGGACCGAACG	58.0	184 bp
*PhTPS4*-Q3′	GCCACGGAATGTGAAGGAAG		
*Ph18S*-Q5′	AGTTAGGGGATCGAAGACGA	55.0	153 bp
*Ph18S*-Q3′	CAGCCTTGCGACCATACTC		

Prior to the activity assay, the eluted protein solution was incubated with the sumo protease (More Biotech, China) to cut off the sumo-His tag, which was at the N-terminal of the target protein. The final working protein solution (50 mM Tris/HCl, 200 mM NaCl, pH 8.0) was obtained using a Ni-agarose column to remove the cleaved sumo-His tag and the sumo protease in the mixture. The target protein concentration in the final solution was determined by a Bio-Rad DC Protein Assay (Hercules, CA, USA) and 10% SDS-PAGE.

### Enzyme activity measurement

The enzyme activity of the TPS domain of PhTPS1–4 was determined in 100 μL of working solution containing 10 mM MgSO_4_, 100 mM UDP-galactose (Sigma Aldrich, Taufkirchen, Germany), and 40 mM G3P (Sigma Aldrich) according to the method of Pade et al. [6] Reaction mixtures were incubated for 16 h at 30 °C and then heated at 100 °C for 5 min to terminate the reaction. The reaction mixture was then treated with 1 U of alkaline phosphatase (CIAP; Fermentas) for 2 h at 37 °C to dephosphorylate the intermediate (iso)floridoside phosphate. After reaction, the mixed solution was extracted, purified, lyophilized, and re-dissolved in 200 μL methanol for HPLC–MS analysis. The enzyme activity corresponded to 1 μmol (iso)floridoside produced in 1 min by 1 mg of protein.

### HPLC–MS/MS analysis

The reaction solution above was analyzed on an UltiMate™ 3000 HPLC system with a Q Exactive hybrid quadrupole-Orbitrap mass spectrometer (Thermo Fisher Scientific, USA) using a Xbridge Amide column (100 mm × 3 mm, 3.5 μm, Waters) at 25 °C. The constant solvent system was 90% acetonitrile (A)–10% water (10 mM CH_3_COONH_4_). The flow rate was 0.3 mL min^− 1^ for 35 min and the injection volume was 10 μL.

The Q Exactive hybrid quadrupole-Orbitrap mass spectrometer was operated in the data dependent mode, automatically switching between full scan MS and MS/MS acquisition with electrospray ionization (ESI) in the negative ionization mode. The mass range was scanned from 50 to 600. The MS/MS parameters were set as follows: Automatic Gain Control (AGC) target 2 × 10^5^; maximum ion time 120 ms; isolation width 4.0 *m*/*z*. The typical mass spectrometric conditions were: a sheath gas pressure (N_2_) flow-rate, 25 L/min; auxiliary gas pressure (N_2_) flow-rate, 5 Abs; spray voltage, 2.5 kV; vaporizer temperature, 300 °C; and capillary temperature, 350 °C; collision gas pressure, 1.5 mTorr.

The quantification of (iso)floridoside was performed on a Finnigan Surveyor and TSQ Quantum Access system (Thermo Fisher Scientific Inc., Pittsburgh, PA, USA), referring to Chen et al. [18]. The calibration curves for (iso)floridoside quantification were constructed with standard compounds extracted directly from *P. haitanensis.*

### Sample treatment

All treatments were performed at a density of 500 mg thalli per 150 mL sterile seawater. For the desiccation treatment, the thalli were subjected to desiccation for 0, 1, 2, 3, and 4 h under 20 °C, 100 μmol·photons·m^− 2^·s^− 1^, and 75% humidity. For the high temperature stress treatment, the thalli were cultured at 35 °C for 30 min and then transferred to 20 °C to recover for 1 and 3 h. For the salt stress treatment, the thalli were cultured in medium supplemented with 500, 700, 100, and 1400 mM NaCl for 1 h under 20 °C. All samples were collected, frozen rapidly in liquid nitrogen, and stored at − 80 °C for RNA isolation. Salt stress-treated samples were processed according to the method of Chen et al. [18] and analyzed by LC–MS.

### Real-time quantitative (qRT) PCR analysis of *PhTPS* under different stresses

The qRT-PCR analysis was performed with SYBR Premix Ex Taq (TaKaRa) on a Mastercycler EP realplex real-time PCR system (Eppendorf, Hamburg, Germany). The specific qRT-PCR primers for *PhTPS1–4* are listed in Table 3. *Ph18S* was used as an internal reference gene. The PCR procedure was as follows: 95 °C for 3 min; 40 cycles of 95 °C for 10 s, Tm°C for 18 s, 72 °C for 15 s, and a dissociation curve analysis to determine target specificity. All reactions were performed in triplicate. Relative gene quantification was performed using the comparative 2^-ΔΔCt^ method and normalized to *Ph18S.*

### Statistical analysis

The data for the qRT-RCR results were obtained from at least three independent biological experiments. LC–MS analysis was performed in biological triplication and technical triplication for validation. Each treatment was evaluated using analysis of variance (ANOVA) in SPSS 22.0 (IBM Corp., Armonk, NY, USA). Comparisons among three groups were made using one-way ANOVA with Tukey’s multiple comparison tests.

## Additional files

Additional file 1: **Figure S1.** Multiple sequence alignments of the deduced amino acid sequences of the trehalose-6-phosphate synthase (TPS) domains for PhTPSs with primary sequences of the TPS domains for other species and GGPS domains for cyanobacteria. The conserved residues are marked by a star. The accession numbers corresponding to the protein sequence of the different species can be searched in Additional file 3: Table S1. * indicates glucose-6-phosphate binding sites, # indicates UDP-glucose binding sites. The light blue background indicates the unique cyanobacteria GGPS protein residues; the yellow frame indicates the low conserved residues of red algae, brown algae, and diatoms. The pink frame indicates the sequences of the four TPS members of *P. haitanensis*. (PDF 655 kb)

Additional file 2: **Figure S2.** The phylogenetic tree of the only TPS and TPP protein are constructed, respectively. (A) A NJ tree was constructed to show the phylogenetic relationships of the TPS proteins using the functional-related amino acid sequences from prokaryotes, red algae, diatoms, brown algae, fungi, green algae, plants, and animals. (B) A NJ tree was constructed to show the phylogenetic relationships of the TPP proteins using the functional-related amino acid sequences from prokaryotes, red algae, diatoms, brown algae, fungi, green algae, plants, and animals. Their accession numbers are indicated in Additional file 3: Table S1. There were 1,000 bootstrap replicates. The red triangle shows PhTPS1–4. The functional domain in each sequence was retrieved using the Conserved Domain tool in NCBI and is marked by a superscript. (PDF 280 kb)

Additional file 3: **Table S1.** TPS and GGPS related genes and proteins in different organisms. (PDF 129 kb)

## Data Availability

The datasets used and/or analysed during the current study are available from the corresponding author on reasonable request.

## Abbreviations

FPS: Floridoside phosphate synthase
G3P: Glycerol-3-phosphate
GalG: Galactosyl glycerol
GGPS: Glucosyl-glycerol-phosphate synthase
TPS: Trehalose-6-phosphate synthase
