# Supplementary material for: Putative trehalose biosynthesis proteins function as differential floridoside-6-phosphate synthases to participate in the abiotic stress response in the red alga Pyropia haitanensis
Source: BMC Plant Biol. 2019 Jul 19;19:325. doi: 10.1186/s12870-019-1928-2 (PMC6642608; doi:10.1186/s12870-019-1928-2)
Supplement: Supplementary file 1 — Figure S1. Multiple sequence alignments of the deduced amino acid sequences of the trehalose-6-phosphate synthase (TPS) domains for PhTPSs with primary sequences of the TPS domains for other species and GGPS domains for cyanobacteria. The conserved residues are marked by a star. The accession numbers corresponding to the protein sequence of the different species can be searched in Additional file 3: Table S1. * indicates glucose-6-phosphate binding sites, # indicates UDP-glucose binding sites. The light blue background indicates the unique cyanobacteria GGPS protein residues; the yellow frame indicates the low conserved residues of red algae, brown algae, and diatoms. The pink frame indicates the sequences of the four TPS members of P. haitanensis. (PDF 655 kb) [file 12870_2019_1928_MOESM1_ESM.pdf]

```
                                                              *                          #
E. coli K12 TPS                     ------------------SRLVVVSNR---------IA-----PPDEHAASAGGLAVGILGALKAAGG----------------------L  37
M. thermoacetophila PT TPS          ------------------MRLVIISNRLPVTVVEEKG-------AIRFMDSVGGLSTGIRSFIASDKARAEMIQD--------------CL  52
A. machipongonensis TPS             -----------------MAKTIIVSNRLPISLRHRNG-------KFEFKPSAGGLATGLDSIYKSGEN----------------------I  45
M. marina ATCC23134 TPS             -----------------MSKTIIVSNRLPIKIQKNNQ-------ELTYTPSAGGLATGLGSIYKEGNN----------------------I  45
S. enterica Virchow str. SL491 TPS  -----------------MSRLVVVSNR---------IA-----PPD-NKGGAGGLAVGVLGALKAAGG----------------------L  37
M. tuberculosis TPS                 -------------ETFGDSDFVVVANRLPVDLER-LPD-----GSTTWKRSPGGLVTALEPVLRRRRG----------------------A  64
C. watsonii WH8501 TPS              -----------------MSKTIIVSNRLPVKIERNQAG------EFEYKTSEGGLATGLGSVYKEGDN----------------------I  46
A. platensis GGPS                   ----------------MKSSLVILYHREPYDEVIENGK-----IHYRAKKSPNGIVPTLKSFFAN-VNKGT--------------------  49
C. sp. ATCC51142 GGPS               ----------------MKSSLVILYHREPYDEVVENGQ-----VRYLPKKSPNGIVPTLKSFFADDNNQGT--------------------  50
S. sp. PCC7002 GGPS                 ----------------MKSSLVILYHREPYDEVRENGK-----TFYRDKTSPNGIMPTLKSFFAN-AEQST--------------------  49
A. sp. CCMEE5410 GGPS               ----------------MKSSLVILYHREPYDEVIENGK-----VHYRPKKSPNGILPTLKSFFAS-VNQGT--------------------  49
C. chthonoplastes PCC7420 GGPS      ----------------MKSSLVILYHREPYDEVVENGK-----VHYRAKKSPNGIVPTLKSFFAG-VNQGT--------------------  49
C. merolae CM2362 TPS               --TRTDMHSEMQSEDESKPKLIIVSNRLPITMKR-RND-----GQYDYTMSSGGLVSALEGALDPRELPFI-------------------- 390
C. merolae 10D TPS                  --ETSAQLTGGVDGAVKPRRLFIVSHRLPVQVHFSPVQ-----NKNKPKIILNSSGLFASLRHLRKEYTIR-------------------- 201
C. merolae CM3596 TPS               PYSGASSLLGTLPPLTNPPTLIIVIYRLPVIAKRS-------ERGWSFSWDDDALYLTSLGLRRGLDG-KIRIL----------------- 263
G. sulphuraria TPS                  ----VICVSLQLPSEGT-----------------------------CSSRSLFQTGLANGIIDFQSMNNLP-------------------- 114
G. sulphuraria TPS-1                PSSRG-----------LPSKLLIVLYRLPVIAKRT-------SNGWSFKWDDDALYLTSVGLRDGLQG-KIRFY----------------- 227
G. sulphuraria TPS-2                ------------------PKLLVVSNRLPVTVTK-DNN-----GAYHFNMSSGGLVSALKGVKS--LMPFL-------------------- 212
P. haitanensis TPS1                 ------------------RRLLVVSNRLPVTINK-NND-----GQWDFKMSAGGLVSALAGVKN--EFPFV-------------------- 172
P. haitanensis TPS2                 --ASRAAKRLPPATRMHSGPLFVASFRLQLSVDISADT-----GAVKASLSAGGLGLVPAFRHLVSRTRIV-------------------- 198
P. haitanensis TPS3                 --SGSGGGGGGNGDGGS-------GDHGNGGG---SNG-----VRYGKGSRSNGIDLDDPVSRSYNPWRDD--------------------  99
P. haitanensis TPS4                 -FGDAI------------ESLVIVLYRLPIIAKRDS-----TTGAWSFKWDDDALYLTSTGLRKGLEQLKVAPL----------------- 223
P. yezoensis TPS-1                  --AQSSLVTLADMERQE--------------------R-----LIIG-QMCELRRKLDDARIRSG--------------------------  80
P. yezoensis TPS-2                  ------------------RRLLVVSNRLPVTISK-NHE-----GQWDFKMSAGGLVSALAGVKN--EFPFV-------------------- 172
P. yezoensis TPS-3                  --ASRAAKRVPPATRMHSGPLFVASFRLQLSVDISADT-----GAVKASLSAGGLGLVPAFRHLVSRTRIV-------------------- 239
P. yezoensis TPS-4                  -FGDAI------------ESLVIVLYRLPIIAKRDA-----TTGAWSFKWDDDALYLTSTGLRKGLEQLKVAPL----------------- 124
P. umbilicalis TPS-1                -FGDSI------------ESLVIVLYRLPIISKRDH-----TTGAWDFKWDDDALYLTSTGLRKGLEQLKVAPL----------------- 232
P. umbilicalis TPS-2                --GGVGVGGGGGGGGGADSSGGSGGGVGGGGGGCYGGG-----WHRGGGSGGGGFDPDDSVSHSFNPWSGE-------------------- 375
C. crispus TPS-1                    --KTGASLLHPKKGRRHQR-LFVASFRLPLTVEIGDD------GEVKSSISNGSLGLFSAFKDLLGRVPIR-------------------- 142
C. crispus TPS-2                    SIGSDQ------------EGLIIVLYRLPVIATCD------PDGKWSFAWDDDALYLTSTGLRRGLEELKIKPL----------------- 156
C. crispus TPS-3                    --TRIISVSFRLPKKEHRESVR-QQFFDRGLPPKAIFS-----LHGASEFFFVWVGTADGQGDVEAHTFEE-------------------- 189
P. tricornutum CCAP1055/1 TPS-1     ------------------DGLVVVSAFLPVVLHRTDE------GKWTADWDYEMLLSMHTH---LRVIRVGVVK----------------- 296
P. tricornutum CCAP1055/1 TPS-2     ------------------NRIFFICFHLPVVVVQSQST-----GQWRASWSESILAKTEGSQILSTYEAYWVGT-----------------  68
T. pseudonana CCMP1335 TPS-1        ------------------DGLIVVSAFLPVHVTRSDN------GEWSADWDYEALLSMQTH---LRVTRVG--------------------  46
T. pseudonana CCMP1335 TPS-2        ------------------ESIFIVCYHLPVILTRDPDD-----STWTACWSESLIAKSETHGVSSTRKTTWIGT-----------------  51
T. pseudonana CCMP1335 TPS-3        ------------------RRIFIVCYHLPVIVSKNPEN-----GSWEACWAESLLAKTSGS-FDPAFEPHRVGT-----------------  72
S. japonica TPS                     -FGDAI------------ESLVIVLYRLPIIAKRDA-----TTGAWSFKWDDDALYLTSTGLRKGLEQLKVAPL----------------- 124
S. japonica TPS-2                   ------------------DGVVVVSFFLPVIIDKDID------GNWVVEWDYESLLSLHSASIDMRVTRVGFLR----------------- 223
S. henslowianum TPS                 -FGDAI------------ESLVIVLYRLPIIAKRDA-----TTGAWSFKWDDDALYLTSTGLRKGLEQLKVAPL----------------- 124
U. pinnatifida TPS                  -FGDAI------------ESLVIVLYRLPIIAKRDA-----TTGAWSFKWDDDALYLTSTGLRKGLEQLKVAPL----------------- 124
C. albicans TPS                     -------------------KVLVVSNRIPVTIKR-LDN-----GSYDYSMSSGGLVTALQGLKK--TTEFQ--------------------  48
C. higginsianum TPS1                -------------------RLLLLSNRLPITIKR-SDD-----GSYTFSMSSGGLVTGLSGLSK--TTSFQ--------------------  56
S. cerevisiae TPS                   -------------------NIIVVSNRLPVTITKNSST-----GQYEYAMSSGGLVTALEGLKK--TYTFK--------------------  61
F. cylindrus CCMP1102 TPS           -------------------KILFICFHLPVVVVKVD-------GKWEATWSESLLASKDSSRIVANYRAHWIGT-----------------  71
V. carteri f. nagariensis TPS-1     -------------------RLIIVSNHLPLRVKRGATG-------WEFEWDEDALVGQAKEG--VPKDLEVLY------------------  52
C. reinhardtii TPS-1                -----------------AARLVVVANRLPVTCSK-DSN-----GRWQLQSSAGGLVSALKGVSN---YVTL-------------------- 111
C. reinhardtii TPS-2                ------------------TRLIIVSNHLPLRVKRATTS-------WEFEWDEDALVGQAKEG--IPSDLEVMY------------------  95
C. reinhardtii TPS-4                -------------------RLIIVSNHLPLRVKRATTS-------WEFEWDEDALVGQAKATGGIPSDLEVMY------------------  53
O. tauri TPS-1                      -----------------RESLIIAANRLPVSVKR-KAD-----GTWDVRPSAGGLVSALLGVRKT--YGMT-------------------- 186
O. tauri TPS-2                      -------------------RVFVVSNTLPLKMREDAAAGKMYGHAYAFEADEESIYDQCREGALKGGQFEVVIN-----------------  99
A. thaliana TPS-3                   -----------------RQTLLVVANRLPASAKR-TGE-----HSWSLEMSPGGKFNLLV-------------------------------  46
A. thaliana TPS-4                   -----------------RPRLLVVSMSLPVTAKR-TGE-----ESWSFTMSPGGLVSALLGLKE---FETK--------------------  47
A. thaliana TPS-5                   ------------------DRIIIVGNQLPIKSHRN------SAGKLSFSWDNDSLLLQLKDG--MREDMEVVY------------------ 106
A. thaliana TPS-8                   ------------------ERKIIVANMLPLQSKRD--A---ETGKWCFNWDEDSLQLQLRDG--FSSETEFLY------------------ 104
Z. mays TPS                         -----------------KQRLLVVANRLPVSANR-RGE-----DHWSLEISAGGLVSALLGVKD---VDAK-------------------- 157
N. tabacum TPS                      -----------------RQRLLVVANRLPVSAVR-RGE-----ESWSLEISGGGLVSALLGVKE---FEAR-------------------- 134
O. sativa TPS                       ------------------ERLIVVANQLPVVARRRPGA---AAGGWAFSWDDDSLLLRLRDG--VPDEMEVLF------------------ 122
Z. marina TPS                       ------------------DRLIIVGNQLPVVAKRR--S---DNAGRDFSWDDESLLLQLKDG--LPDDMEVLY------------------ 114
P. patens TPS                       -----------------KQRLLVVANRLPVSAIR-LEG-----DKWDLQLSAGGLVSALLGVKHS--FETR--------------------  91
P. patens TPS-X1                    -----------------AQRLLVVANRLPVSATR-LDG-----DKWDLQLSAGGLVSALLGVKQI--FETR-------------------- 210
C. elegans TPS-1                    --EEPHTQSPLVDMSHDKQRVINVSNAPPVSISRKTSG------SWEIKQGSGGLVACVDPVMSADKKNIWLSNLGVNMQEELKEHSTSTN 390
H. sapiens TPS                      --WSPDTLRKILADDLPETQVIVVSNREPYIHN--IKD-----DSVELLVPASGLVSALEPITRACAG----------------------T 132
F. chinensis TPS                    -------MVIN-----STSPMVVVANRLPFTVQKNPVT-----GQMERKHCAGGLVTAVAPVVVETEG----------------------L  52
C. floridanus TPS                   -------MSIEPGSFTSNGSMIVVSNRLPFVLKRNELT-----GQLERKASAGGLVTAVAPVVISGNG----------------------I  57
B. antarctica TPS                   --RCEPEITITAADSPSKRSLIVVSNRLPFVLKK-AAD-----GRMTRLASAGGLVTAVAPVVIKGKG----------------------L  69
```

```
                                                                                                       *         *
E. coli K12 TPS                     WFGWSGETGNE----------DQPLKK------VKKGNITWASFNLSEQD-------------LDEYYN-QFSNAVLWPAFHYRL------  92
M. thermoacetophila PT TPS          WVGWPGVDLKR------------R-NIQDITSQLKDMN--CWPVFFSNRV-------------IDKFYH-GFCNRTIWPLFHYFP------ 108
A. machipongonensis TPS             WIGWPGNTVDD------------AEQRAEIIIELHALK--MAPVFLSKED-------------VEQFYE-GFSNETLWPAFHYFT------ 102
M. marina ATCC23134 TPS             WLGWAGLHLTS------------EAEKNQVTEDLEKES--LKPIFLSEED-------------VEGFYE-GFSNATIWPSFHYFP------ 102
S. enterica Virchow str. SL491 TPS  WFGWSGETGNE----------DEPLKK------VTKGNITWASFNLSEQD-------------YEDYYC-QFSNAVLWPAFHYRL------  92
M. tuberculosis TPS                 WVGWPGVNDDG----------AEPDLH-VLDGPIIQDELELHPVRLSTTD-------------IAQYYE-GFSNATLWPLYHDVI------ 124
C. watsonii WH8501 TPS              WVGWPGLAVNK------------TEDKEEICSRLKESN--MSPVFLTKNE-------------IEEYYE-GFSNETLWPNFHYFN------ 103
A. platensis GGPS                   WVAWKQVTAKQ------------KADFQERVEVEDEGNYTVRRIALNADQ-------------VKHFYH-ITSKEAFWPVLHSFP------ 108
C. sp. ATCC51142 GGPS               WIAWKQVSPEQ------------KNQFNKNVVVEDDGNYRVSRIPLTAEQ-------------VRDFYH-VTSKEAFWPILHSFP------ 109
S. sp. PCC7002 GGPS                 WVAWKQISGKQ------------QENFQAKMAFPGQENSVVHRIPLSADQ-------------VKNFYH-ITSKEAFWPILHSFP------ 108
A. sp. CCMEE5410 GGPS               WIAWKQVTAKQ------------RSNFETLVTVEGEGNYNVCRIPLSADQ-------------VKHFYH-ITSKEAFWPILHSFP------ 108
C. chthonoplastes PCC7420 GGPS      WIAWKQVNAKQ------------KANFEQRIVVEGEANYNVRRIPLSADQ-------------VKHFYH-ITSKEAFWPILHSFP------ 108
C. merolae CM2362 TPS               WVGWPG--------------GEVDPADETQVSTELLHRYGLVPVFLSAEL-------------HELYYN-GFCNDVLWPLFHYVPLQVV-- 451
C. merolae 10D TPS                  WIGQL----MQIQQLS--------AEQQDITRRILLAEHGFIPLFIDAK---------------LCRMHQIFCNSVLWRIFHYIP------ 259
C. merolae CM3596 TPS               WVGVLNC------------DDEVPIHEQSHVSNTLYQRFGCIPVFLPTE--------------TRMKFYQGFCKGVLWPLFHMITEIMD-- 326
G. sulphuraria TPS                  FAFVGC-------APS-----TTTVGSPNATEKRRLHGEQFFPVLLPQE---------------SFEQYFSFCRQILWNLLHYNFS----- 173
G. sulphuraria TPS-1                WIGILNC------------EDDIPEDEEEGVTQTLMDRFSCIPVFVPKA--------------TLTKFYQGFCKGVLWPLFHMIIQVTD-- 290
G. sulphuraria TPS-2                WIGWPG--------------TEIDPNERDYVRNKLREGYSCFPVFITDEQ-------------ANLYYN-GFCNDVLWPLFHYVPLPIV-- 273
P. haitanensis TPS1                 WVGWSG--------------SEVPDEDQDSLRTELRAQHECVPVFLSDFD-------------AHLYYN-GFCNDVLWPLFHYVPLPIV-- 233
P. haitanensis TPS2                 WLGMP---VFAAGQVP--NAATQARIQARLRARKPSAVLSYAPIFPRPS---------------DAVTHQAFCNNVLWALFHYLP------ 263
P. haitanensis TPS3                 LSSSQR-------RLG-----SGRRPPRGSRGKDPADRSRYVAVTLPSS---------------LEAPFYAFCEETLWRLLHYDYG----- 158
P. haitanensis TPS4                 WVGILNS------------EDEVPRQEREGVADRLLEEFNCVPVFIPHD--------------TLKHFYQGFCKGTLWPLFHMVSSATD-- 286
P. yezoensis TPS-1                  -VKRSR-------RPK-------RRGAALSSDGLSAEIATHDLLS-PVN---------------VAVPGERIAAITAAAAVTAVA------ 135
P. yezoensis TPS-2                  WVGWSG--------------SEVPDADQDSLRTELRAQHECVPVFLSDFD-------------AHLYYN-GFCNDVLWPLFHYVPLPIV-- 233
P. yezoensis TPS-3                  WLGMP---VFAAGQVP--NEATQARIQARLRARKPSAVLSYAPIFPRPS---------------DAVTHQAFCNNVLWALFHYLP------ 304
P. yezoensis TPS-4                  WVGILNS------------DDEVPRQEREGVADRLLEEFNCVPVFIPHD--------------TLKQLYQGFCKGTLWPLFHMVSSATD-- 187
P. umbilicalis TPS-1                WVGILNS------------DEEVPMRERDGVADRLLEEFNCVPVFIPHD--------------TLKQFYEGFCKGVLWPLFHMVSTATD-- 295
P. umbilicalis TPS-2                PSICGR-------RPRGGGGSSGRRLPRGSRGKDPAERSRHVAVTLPSS---------------LEAPFHAFCEETLWRLLHYDYG----- 439
C. crispus TPS-1                    WLGAPGQTFHDAAQLPPDERSKMERHFQKRRHRKSMGLLSYVPLFPDAE---------------DAAAHQEFCNSVMWPLFHYIP------ 212
C. crispus TPS-2                    WIGIINT------------DTPIPPADRDHISRVLLDDFNAVPVYLPQE--------------TRTKFYQGFCKDTLWPAFHMLTNVS--- 218
C. crispus TPS-3                    VYGEEH-------RTG------WRSLNSRHPDVQKVKRSRYVAVSLPDEP-------------HLLANYDTFCEDALWPLLHYDYA----- 249
P. tricornutum CCAP1055/1 TPS-1     WRGWHGRSGKNG------SPENGVPRSERKLVEECLQNFDCVPVWIEPL--------------LFGEFYNGFCKGVLWPVVHNVTSVYSNR 367
P. tricornutum CCAP1055/1 TPS-2     VTTSPP----------------LTNEKDKDEVRQILQEMNCIPIILDPE--------------VRQAHYYGFCKQVLWPAFHNIDLLDLST 129
T. pseudonana CCMP1335 TPS-1        --------------------TSGVPVDERHKVEAVLRPFNCVPVWVPTS--------------LFGEMYNGFCKGVLWPILHN--------  95
T. pseudonana CCMP1335 TPS-2        VSNIPAQ--------------FLADENEREAIRVVLKDMDCIPIFFVEEGA----------ESVSELHYLGFCKQVLWPSFHNL------- 111
T. pseudonana CCMP1335 TPS-3        VTTNTP----------------ITSEADKEALSSLLATMNCTVLFFDDA--------------IRDAHYKGFCKQVLWLAFHHVDILDMHD 133
S. japonica TPS                     WVGILNS------------DDEVPRQEREGVADRLLEEFNCVPVFIPHD--------------TLKQFYQGFCKGTLWPLFHMVSSATD-- 187
S. japonica TPS-2                   -------------------YADGVKPEEEEAVAKALLPFSCVPVFLERS--------------LAQKFYRDFCKGVLWPVFHHVVDVYG-- 279
S. henslowianum TPS                 WVGILNS------------DDEVPRQEREGVADRLLEEFNCVPVFIPHD--------------TLKQFYQGFCKGTLWPLFHMVSSATD-- 187
U. pinnatifida TPS                  WVGILNS------------DDEVPRQEREGVADRLLEEFNCVPVFIPHD--------------TLKQFYQGFCKGTLWPLFHMVSSATD-- 187
C. albicans TPS                     WYGWPG--------------LEIPEDEQTKVNDELKSKFNCTAIFLSDTI-------------ADLHYN-GFSNSILWPLFHYHP------ 105
C. higginsianum TPS1                WYGWPG--------------LEVPENEIAGMKQRLKDEYDAHPVFIDDEL-------------ADKHYN-GFSNSILWPLFHYHP------ 113
S. cerevisiae TPS                   WFGWPG--------------LEIPDDEKDQVRKDLLEKFNAVPIFLSDEI-------------ADLHYN-GFSNSILWPLFHYHP------ 118
F. cylindrus CCMP1102 TPS           VTAHPP----------------ISKEEDRQAIRAILAEMECTPIFLDPS--------------TAQAHYYGFCKQVLWPAFHNIDLLDLST 132
V. carteri f. nagariensis TPS-1     -VGSLP--------------VDVALEEQDAVAAQLKRLYNCCPVFLDKD--------------IRDKFYKGCCKQQLWPLFHYVLPVS--- 111
C. reinhardtii TPS-1                WIGWPG--------------IWVKAGRERDELTAMLKSEGYCPVWMDHTL-------------LDLYYN-GFCNSVLWQLFHYVPLNID-- 172
C. reinhardtii TPS-2                -VGSLP--------------VDVALEEQDAVAAQLKRLYNCCPVFMEKE--------------LKEKFYKGCCKQQMWPLFHYVLPMS--- 154
C. reinhardtii TPS-4                -VGSLP--------------VDVALEEQDAVAAQLKRLYNCCPVFMEKE--------------LKEKFYKGCCKQQMWPLFHYVLPMS--- 112
O. tauri TPS-1                      WIGWPG--------------VFIEPGPERDSLTATLRRQNLLPVYLTKSQ-------------VELYYN-GYCNNVLWSLFHYVPLNFE-- 247
O. tauri TPS-2                      -VGQLP--------------MEVPMEMQDAVANDLERRYNCMPVFLPKE--------------VKENFYNGFCKQYMWPLMHYVLPMS--- 158
A. thaliana TPS-3                   ---------------------------EKDAVSKSLAEMKCIPVFLN-EV-------------FDQYYN-GYSNGILWPILHHMGLPQE--  93
A. thaliana TPS-4                   WIGWPG--------------VDVHDAIGKKTLSITLAEKGCIPVFLE-EV-------------CDQYYN-GYCNNILWPIFHYLGTPPE-- 107
A. thaliana TPS-5                   -IGCLK--------------EQIDTVEQDDVSQRLLENFKCVPAYIPPE--------------LFTKYYHGFCKQHLWPLFHYMLPLT--- 165
A. thaliana TPS-8                   -VGSLN--------------VDIETNEQEEVSQKLLEEFNCVATFLSQE--------------LQEMFYLGFCKHQLWPLFHYMLPMF--- 163
Z. mays TPS                         WIGWAG--------------VNVPDEVGQRALTKALAEKRCIPVFLDEEI-------------VHQYYN-GYCNNILWPLFHYLGLPQE-- 218
N. tabacum TPS                      WIGWAG--------------VNVPDEAGQRALTKALAEKRCIPVFLDEEI-------------VHQYYN-GYCNNILWPLFHYLGLPQE-- 195
O. sativa TPS                       -IGTLR--------------ADVPACEQDEVSQSLIDGFGCAPVFLPAG--------------LYDRFYQHFCKGYLWPLFHYMLPFASAL 184
Z. marina TPS                       -VGCLR--------------VIVDPEEQDDVSQTLLEKFKCVPAFLTEE--------------ILEKYYHGFCKKLLWPLFHYMLPLT--- 173
P. patens TPS                       WIGWPG--------------VSVHDEKGRASLTEALALKGCVPVFLDDDT-------------VDQYYN-GYCNNVLWPLFHYIGLPQA-- 152
P. patens TPS-X1                    WIGWAG--------------VSVHDERGRASLTEALALKGCVPVFLDDDT-------------VDQYYN-GYCNNVLWPLFHYIGLPQA-- 271
C. elegans TPS-1                    SLGLPLIKQACAGEVFCVLERNEKKEELTPKQQAVESDMSLLSVLNTYNKHSYQLNPVVVNQDDYNTYYGGISNGLLWPALHNLPQYIS-- 479
H. sapiens TPS                      WIAYGGGTADR----------LTVDKNDRIEVPPGNPSYALRRVWLTEEE-------------YQGYYL-GFANEGLWPLCHIAF------ 193
F. chinensis TPS                    WVGWSGQHQED------GVVEIPEANPNDQSPTAGLKSWQVKPMMSQKTL-------------FDNYYN-GCCNATFWPLFHSMP------ 117
C. floridanus TPS                   WVGWPGMHMEN------PNEPIPESDPNDRTPTAGLLSRKVVAVHVDAGI-------------FDSYYN-GCCNGTFWPLFHSMP------ 122
B. antarctica TPS                   WVGWPGLHLPKD----FNDANIPESDPSDQSPTAGLKSEQVVTVNCDPDL-------------FEMYYN-GCCNGTHWPLFHSMP------ 136
```

E. coli K12 [TPS]	---------------------------------DLVQFQRPAWDGYLRVNALLADKLLPLLQDD---------------------------	123
M. thermoacetophila PT [TPS]	---------------------------------SLTSYNKDHWNTYIHANRVFAEEISRILMPG---------------------------	139
A. machipongonensis [TPS]	---------------------------------QYMIYNPDHWDAYVRVNQKFCEAIVKKADPD---------------------------	133
M. marina ATCC23134 [TPS]	---------------------------------QYAVYQDIFWESYQQVNQKFAEAILEVCEAN---------------------------	133
S. enterica Virchow str. SL491 [TPS]	---------------------------------DLVQFQRPAWEGYMRVNALLADKLLPLIKEN---------------------------	123
M. tuberculosis [TPS]	---------------------------------VKPLYHREWWDRYVDVNQRFAEAASRAAAHG---------------------------	155
C. watsonii WH8501 [TPS]	---------------------------------QYAVYSDVFWNTYKKVNKKFAKKLEEIIEDG---------------------------	134
A. platensis [GGPS]	---------------------------------YHFTSETADWENFTTINRLFAEAACEEAADD---------------------------	139
C. sp. ATCC51142 [GGPS]	---------------------------------YHFTSESSNWDNFVKINRLFAEAACEEAADD---------------------------	140
S. sp. PCC7002 [GGPS]	---------------------------------WQFTYDSSDWENFKQINEMFAEAACEDADDD---------------------------	139
A. sp. CCMEE5410 [GGPS]	---------------------------------YHFTYESSDWENFHTINRLFAEAACEQAADD---------------------------	139
C. chthonoplastes PCC7420 [GGPS]	---------------------------------YHFTYETSDWENFHTINRLFAEAACDEAADD---------------------------	139
C. merolae CM2362 [TPS]	----------------------------SQDGERKFD--YKYWAAYTTANQRFAEAIMSVYRRG---------------------------	485
C. merolae 10D [TPS]	-----------------------------VGVEGERAFCSELWSAYCRVNALYAEAISRESQPG---------------------------	294
C. merolae CM3596 [TPS]	-----------------------------EEKMETKKFDRSLWHVYCTVNRKFADTVVAVYH-----------------------------	359
G. sulphuraria [TPS]	----------------------------KLLSSDA-FELHRGWEVYREVNIQFAEAVCEVYEDG---------------------------	208
G. sulphuraria [TPS-1]	-----------------------------DG-GRTKQFDRSLWHVYCLVNRKFADVVVGVYH-----------------------------	322
G. sulphuraria [TPS-2]	----------------------------SSDGERKFD--FKYWQAYSAANYRFAEAVLQVYQEG---------------------------	307
P. haitanensis [TPS1]	----------------------------SSDGERKFD--VKYWEAYSKANHRFAEAIMQVYEPG---------------------------	267
P. haitanensis [TPS2]	-----------------------------LSFEGDRSFRPEMFEAYKRVNEEYALALLREFERSRRGVPPGHGLGLGGRPASAKTSVDAQA	325
P. haitanensis [TPS3]	----------------------------ALGNGGSGGVDTRDWEAYQAVNRRFAEAVTEVYEEG---------------------------	194
P. haitanensis [TPS4]	-----------------------------HTEHTTR-FDDHLWRVYMNVNRMFRDKVVEVYDG----------------------------	319
P. yezoensis [TPS-1]	----------------------------VAGVVAAATAVVAAVTTAAAMVAMAFEAVTEVYEEG---------------------------	171
P. yezoensis [TPS-2]	----------------------------SSDGERKFD--VKYWEAYSKANHRFAEAIMQVYEPG---------------------------	267
P. yezoensis [TPS-3]	-----------------------------LSFEGDRSFRPEMFEAYKRVNEEYALALLREFERSRRGMPPGQDPGPTGR------------	354
P. yezoensis [TPS-4]	-----------------------------HTEHTTR-FDDRLWRVYMNVNRMFRDKVVEVYDG----------------------------	220
P. umbilicalis [TPS-1]	-----------------------------HTQHTTR-FDDRLWRVYMNVNRMFRDKVVEVYDG----------------------------	328
P. umbilicalis [TPS-2]	----------------------------SLGNGGSGGVDTRDWDAYQAVNRRFAEAVTEVYEEG---------------------------	475
C. crispus [TPS-1]	-----------------------------LNL-GERTYHVEMFDAYERVNRFYADALIQEWKRT---------------------------	246
C. crispus [TPS-2]	-----------------------------TDGHTKR-FDQESWHAYQRVNRTFSNIIVEHY------------------------------	249
C. crispus [TPS-3]	----------------------------AFGDPDN---LDEAWNAYRFVNKKFAEAISEIYEEG---------------------------	282
P. tricornutum CCAP1055/1 [TPS-1]	PEDNEIFSGQRVVQDKTQFSEYSMDDVEQGPVHGGRGREAALWSAYNTVNRQFADVVQCFN------------------------------	429
P. tricornutum CCAP1055/1 [TPS-2]	CGW--------------------LSDQESGSSDWDQSRLDSWWNSFVSVNQEFCNVITSLSR-----------------------------	171
T. pseudonana CCMP1335 [TPS-1]	-----------------------------GPIHGDGGREAELWAAFTAVNRYFTDVIIQCFN-----------------------------	128
T. pseudonana CCMP1335 [TPS-2]	------------------------------QSDWG-DRLDRWWNAYQFVNRKFSKVVAELVE-----------------------------	142
T. pseudonana CCMP1335 [TPS-3]	------------------------------PATWDQSQIGPWWEAYNIVNRTFAVEVAKMVG-----------------------------	165
S. japonica [TPS]	-----------------------------HTEHTTR-FDDRLWRVYMNVNRMFRDKVVEVYDG----------------------------	220
S. japonica [TPS-2]	---------------------------DQVMRFFAQDVLADLWQAYANVNRRFRDKIVEVYN-----------------------------	164
S. henslowianum [TPS]	-----------------------------HTEHTTR-FDDRLWRVYMNVNRMFRDKVVEVYDG----------------------------	220
U. pinnatifida [TPS]	-----------------------------HTEHTTR-FDDRLWRVYMNVNRMFRDKVVEVYDG----------------------------	220
C. albicans [TPS]	-------------------------------GEMNFD--ENAWAAYIEANKKFALEIVKQVNDD---------------------------	136
C. higginsianum [TPS1]	-------------------------------GEITFD--ESAWAAYREVNRLFAKAVVQDVQDG---------------------------	144
S. cerevisiae [TPS]	-------------------------------GEINFD--ENAWLAYNEANQTFTNEIAKTMNHN---------------------------	149
F. cylindrus CCMP1102 [TPS]	SG----------------------SDG-VATSDWDQSRLDHWWQAYQQVNQEFANVVKGIVERDAVTM-----------------------	177
V. carteri f. nagariensis [TPS-1]	------------------------------PDSSGR-FDQEMWQSYVKANKVFCEKVVEESAT----------------------------	143
C. reinhardtii [TPS-1]	----------------------------SWQKMAEHRAMQMQWQAYQVANEKFADVVLEEYSSQ---------------------------	208
C. reinhardtii [TPS-2]	------------------------------PESSGR-FDQEMWQAYVKANKVFCEKVVEESAT----------------------------	186
C. reinhardtii [TPS-4]	------------------------------PESSGR-FDQEMWQAYVKANKAEAGDTVSCGWR----------------------------	144
O. tauri [TPS-1]	----------------------------ARLNQETN--MTAQWVAYKEANRVFADAIMSVYRDG---------------------------	281
O. tauri [TPS-2]	------------------------------P-MDAR-FETHQWQAYIASNKRFADKLMETVSS----------------------------	189
A. thaliana [TPS-3]	----------------------------YDHDTIKT--FETQYDAYKKANRMFLDVIKENYKDG---------------------------	127
A. thaliana [TPS-4]	----------------------------YRNDATIT--YQSQYEAYKKANQIFFDVVKEHYEEG---------------------------	141
A. thaliana [TPS-5]	------------------------------PDLGGR-FDRSLWQAYLSVNKIFADKVMEVISP----------------------------	197
A. thaliana [TPS-8]	------------------------------PDHGDR-FDRRLWQAYVSANKIFSDRVMEVINP----------------------------	195
Z. mays [TPS]	----------------------------DRLATTRN--FESQFDAYKRANQMFADVVYEHYQDG---------------------------	252
N. tabacum [TPS]	----------------------------DRLATTRS--FQSQFAAYKKANQMFADVVTEHYEEG---------------------------	229
O. sativa [TPS]	PA---------------------------AASGDGR-FDRGAWEAYVLANKYFFEKVVEVINP----------------------------	219
Z. marina [TPS]	------------------------------KDHGGR-FDRSLWEAYVAVNKIFSQKVVEIISP----------------------------	205
P. patens [TPS]	----------------------------DRLAATRS--LDSQFQAYQHANSLFAKIVFSQYQEG---------------------------	186
P. patens [TPS-X1]	----------------------------DRLAATRS--LDSQYAAYQHANKLFAQVVFSQYQEG---------------------------	305
C. elegans [TPS-1]	------------------------------PCFDDPELLREQWCAYVRVNYLFAINAARNSRAQ---------------------------	513
H. sapiens [TPS]	---------------------------------TRPIFRASDWEAYEAVNRKFADTVVAEARNER--------------------------	225
F. chinensis [TPS]	---------------------------------DRAIFQQDKWEAYREVNEQFAKLTVEAVQSLSKSNPE---------------------	154
C. floridanus [TPS]	---------------------------------DRTTFIAEHWRAYSTVNEEFAAKTVDALEQIHKEQENQS-------------------	161
B. antarctica [TPS]	---------------------------------GRTIFSADHWRAYVKVNKLFAEKTVDALERCSQNKGN---------------------	173

E. coli K12 TPS	DIIWIHDYHLL	134
M. thermoacetophila PT TPS	DLVWIHDYHLM	150
A. machipongonensis TPS	DTIWIHDYQLM	144
M. marina ATCC23134 TPS	DTVWIHDYQLL	144
S. enterica Virchow str. SL491 TPS	DIIWVHDYHLL	134
M. tuberculosis TPS	ATVWVQDYQLQ	166
C. watsonii WH8501 TPS	DKIWIHDYQLL	145
A. platensis GGPS	ALIWIHDYNLW	150
C. sp. ATCC51142 GGPS	ALIWVHDYNLW	151
S. sp. PCC7002 GGPS	ALFWVHDYNLW	150
A. sp. CCMEE5410 GGPS	ALIWIHDYNLW	150
C. chthonoplastes PCC7420 GGPS	ALIWVHDYNLW	150
C. merolae CM2362 TPS	DSVWVQDYHLM	496
C. merolae 10D TPS	DLIWVHDFQLM	305
C. merolae CM3596 TPS	EHDLIWIHDYHLM	372
G. sulphuraria TPS	DMIWIHDYHFM	219
G. sulphuraria TPS-1	ESDLIWIHDYHLM	335
G. sulphuraria TPS-2	DYIWVQDYHLL	318
P. haitanensis TPS1	DLIWVQDYHLM	278
P. haitanensis TPS2	GPSNAKTSVDAQAEDVDASDVLLADDLL--AGGAAADAEAANAEPAACNPAAAPAAEPAPASGPGLAGGGREAGGGVEETGVFWVHDFQLM	414
P. haitanensis TPS3	DLVWVHNYHLL	205
P. haitanensis TPS4	DRTLIWVHDYHLM	332
P. yezoensis TPS-1	DLVWVHNYHLL	182
P. yezoensis TPS-2	DLIWVQDYHLM	278
P. yezoensis TPS-3	-PANAKAAAAAPAVDVDAADVLLADVLLPSADGAAASAETVGSETEAGNPAAAPAAEPAPGSGAGPAGGGREAGGGGEENGVFWVHDFQLM	444
P. yezoensis TPS-4	DRTLIWVHDYHLM	233
P. umbilicalis TPS-1	DRQLIWVHDYHLM	341
P. umbilicalis TPS-2	DLVWVHNYHLL	486
C. crispus TPS-1	GIDESEAMFWIHDFHLC	263
C. crispus TPS-2	EKHLIWVHDYHLM	262
C. crispus TPS-3	DLIWVHNYHLM	293
P. tricornutum CCAP1055/1 TPS-1	GHELVWIHGFHLL	442
P. tricornutum CCAP1055/1 TPS-2	PGDIMWIHDYHLS	184
T. pseudonana CCMP1335 TPS-1	EGDLIWIHGFHLM	141
T. pseudonana CCMP1335 TPS-2	GGDIVWVHDYHLA	155
T. pseudonana CCMP1335 TPS-3	IGDVVWVHDYHLS	178
S. japonica TPS	DRALIWVHDYHLM	233
S. japonica TPS-2	EGDMVWVHGFHLL	327
S. henslowianum TPS	DRALIWVHDYHLM	233
U. pinnatifida TPS	DRALIWVHDYHLM	233
C. albicans TPS	DMIWVHDYHLM	147
C. higginsianum TPS1	DLIWVHDYHLM	155
S. cerevisiae TPS	DLIWVHDYHMM	160
F. cylindrus CCMP1102 TPS	IDKTYLWIHDYHLA	191
V. carteri f. nagariensis TPS-1	DTDYVWIHDYHLL	156
C. reinhardtii TPS-1	DVVWVQDYHLM	219
C. reinhardtii TPS-2	DTDYVWIHDYHLL	199
C. reinhardtii TPS-4	GTWAVGSPHAHTL	157
O. tauri TPS-1	DIVWAHDYHLM	292
O. tauri TPS-2	DDDLVWVNDYHLM	202
A. thaliana TPS-3	DIVWCQDYHLM	138
A. thaliana TPS-4	DVVWCHDYHVM	152
A. thaliana TPS-5	DDDFVWVHDYHLM	210
A. thaliana TPS-8	EDDYVWIQDYHLM	208
Z. mays TPS	DVIWCHDYHLM	263
N. tabacum TPS	DVVWCHDYHLM	240
O. sativa TPS	EDDYVWVHDYHLM	232
Z. marina TPS	EDDYVWIHDYHLM	218
P. patens TPS	DVVWCHDYHLM	197
P. patens TPS-X1	DVVWCHDYHLM	316
C. elegans TPS-1	DFIWIHDYHLM	524
H. sapiens TPS	PIVLVQDYHFA	236
F. chinensis TPS	TIPLVWLHDYHLM	167
C. floridanus TPS	NGTPLVWVHDYHLM	175
B. antarctica TPS	NGVPIVWIHDYHLM	187

```
                                                    #
E. coli K12 TPS                         PFAHELRKRG------VNNRIGFFLHIPFPTPEIFNALP--TYDTLLEQLCDYDLLGFQTENDRLAFLDCLSNLT-RVTTRSAKSHTA---  213
M. thermoacetophila PT TPS              LLPQMLRERF------HDLPIGFFLHIPFPSYEVFQILPKDWQRAIVDGLLGSNLIGFHTYDYARNFLRAALRIV-GAESH----------  224
A. machipongonensis TPS                 LLPQMLREIL------PEATIAFFQHIPFPSYEIIRMIP--WRKEILEGVCGADLIGFHTYDDMRHFLSAVGRIT-GMSGQ----------  216
M. marina ATCC23134 TPS                 LVPQLIREKR------PHISIGYFQHIPFPSYEIFRLLP--WRRELLLGVLGADLVGFHTYDDMRHFLSSVNRLA-GIGYF----------  216
S. enterica Virchow str. SL491 TPS      PFASELRKRG------VNNRIGFFLHIPFPTPEIFNALP--PHDELLEQLCDFDLLGFQTENDRLAFLDSLSSQT-RVTTRSGKQHIA---  213
M. tuberculosis TPS                     LVPKMLRMLR------PDLTIGFFLHIPFPPVELFMQMP--WRTEIIQGLLGADLVGFHLPGGAQNFLILSRRLV-GTDTSRGTVGVRS--  246
C. watsonii WH8501 TPS                  VLPAMIRETH------PNSSIGFFLHIPFPSYESFRLLP--WRTDLLTGMLGADFIGFHTYDYVRHFLSSVNRLV-GITDN----------  217
A. platensis GGPS                       LAARYIREMK------PDARIAFFHHTPFPSVDVFNILP--WRQEIVESLLCCDLVGFHIPRYSENFVNVARSLC-EIKVVKREPVAPH-L  231
C. sp. ATCC51142 GGPS                   LAPQFIREKK------PDARIAFFHHTPFPSVDIFNILP--WREAIVDSLLCCDVVGFHIPRYSENFVNVARSLR-PIDIVEKTKVTGH-I  232
S. sp. PCC7002 GGPS                     LTPYFIRQKK------PNAKIAFFHHTPFPSVDIFNILP--WREAIVDSLLCCDLCGFHLPRYVQNFVAVARSLR-KVEITRQVPVDEHAF  232
A. sp. CCMEE5410 GGPS                   MAPHYIRQLK------PNAKIAFFHHTPFPSVDIFNILP--WREEIVDSLLCCDIVGFHIPRYSENFVNVARSLR-PVEVVERGPIPEH-I  231
C. chthonoplastes PCC7420 GGPS          LVPYYIRQLK------PNARIAFFHHTPFPSVDIFNILP--WREEITDSLLCCDIVGFHIPRYSENFVNVARSLR-PVEIVKKEPVPEH-I  231
C. merolae CM2362 TPS                   LLPALLRRKLR-----HSTRIGFFLHTPFPSSEVYRILP--VRREILEGVLAADLIGFHTFDYARHFLSVCTRIL-GLESSHKGVAFH---  576
C. merolae 10D TPS                      LVPQLLRSRQA------DARIGFFLHTPFPSSEIYRMLP--VRRPLLEGMLASDLIGFHTYDYARHFFSVCSRLL-GLETRPNGIDNR---  384
C. merolae CM3596 TPS                   VLPSQLRRKLS------GAKIGFFLHTPWPSSEIYRMLP--VRDELLRGLLSSSLIGFHLFDYARHFLSACVRLLNLEHES-----SRGAL  450
G. sulphuraria TPS                      LLPEMIRSRLW------YAKIGIFLYTPFPSFEVFRSFP--WRTELLSGLLGSSLVGFHNYDSSRHFSESCSRLL-GTEGTPKGIEVRS--  299
G. sulphuraria TPS-1                    VLPSQLRRKLS------GAKIGFYLHIPWPSSEIYRSLP--VRDELLRGLLSASLLGFHLFDYARHFLSACTRLLNLEHES-----RRGSL  413
G. sulphuraria TPS-2                    LLPSLLRRRLR-----DVK-IGFFLHIPFPTSEVYRIIP--VRKELLQGVLAADLIGFHTYDYARHFLSVCTRIL-GLDSSPKGVNYH---  397
P. haitanensis TPS1                     LLPSLLRKRIR-----DVT-IGFFLHTPFPSSEVYRILP--MRNKVLQGVLAADLIGFHTYDYARHFLSVCTRIL-GLEASPKGVDYK---  357
P. haitanensis TPS2                     LVPKMLRERMP------HAKIGFFLHTPFPAGELYRTLP--PRRELLEGMLGADLIGFHTYDYARHFLSACERIL-GLDIRPNCVDNH---  493
P. haitanensis TPS3                     LLPAMLRRRLW------YAKIGFFLYTPFPSSEIFRLLP--QRSEVLRGILGADLAGFHTYDYSKQFLASCARLL-GLEGSPKGIQVE---  284
P. haitanensis TPS4                     LLPQALRSRLS------GVKIGFFLHIPWPSSEVYRVLP--WRNELLKGMLSATLLGFHLFDYARHFLSACVRLLNLEHEA-----NRGSL  410
P. yezoensis TPS-1                      LLPAMLRRRLW------YAKIGFFLYTPFPSSEIFRLLP--QRSQVLRGILGADLAGFHTYDYSKQFLASCARLL-GLEGSPKGIQVE---  261
P. yezoensis TPS-2                      LLPSLLRKRIR-----DVT-IGFFLHTPFPSSEVYRILP--MRNKVLQGVLAADLIGFHTYDYARHFLSVCTRIL-GLEASPKGVDYK---  357
P. yezoensis TPS-3                      LVPKMLRERMP------HAKIGFFLHTPFPAGELYRTLP--PRRELLEGMLGADLIGFHTYDYARHFLSACERIL-GLDIRPNCVDNH---  523
P. yezoensis TPS-4                      LLPQALRSRLS------GVKIGFFLHIPWPSSEVYRVLP--WRNELLKGMLSATLLGFHLFDYARHFLSACVRLLNLEHEA-----NRGSL  311
P. umbilicalis TPS-1                    LLPQALRSRLS------GVKIGFFLHIPWPSSEVYRVLP--WRNELLKGMLSATLLGFHLFDYARHFLSACVRLLNLEHEA-----NRGSL  419
P. umbilicalis TPS-2                    LLPAMLRRRLW------YAKIGFFLYTPFPSSEIFRLLP--QRSEVLRGVLGADLAGFHTYDYSKQFLASCARLL-GLEGSPKGIQVE---  565
C. crispus TPS-1                        LVPKILRDRLP------NARIGYFLHTPFPAGEIFRALA--PRKELLEGLLGADLLGFHTYDYARHFLSACERLL-GVTVQPDHVDNK---  342
C. crispus TPS-2                        LLPYYLRIKLS------GVKIGLYLHIPWPSSEIFRMMP--VRNEILKGLLSATMLGFHLFDYARHFLSACVRLLNLEHEA-----RRGSL  340
C. crispus TPS-3                        LLPSLLRESLW------YAKIGFFLYTPFPSAEIFRIFP--HRVELLRGVIGADLIAFLSYDYSRQFVSSCTRLL-GLEGTPNAIEAD---  372
P. tricornutum CCAP1055/1 TPS-1         ILPSYLTRRIP------MAKVGIFLHTPFPSSEIFRTLW--CREDLLRGMLNADQVGFHLFEYARHFLTSCRRLLGLNYGMIPDASGGHTL  525
P. tricornutum CCAP1055/1 TPS-2         LLAQQLTQAEIGKYGWKMTRKVFFLHIPFPTSQIFRELE--CGERILQGMLHADVVGFHAFDHARHFLNAAKRILGLNYES----LAGGLI  269
T. pseudonana CCMP1335 TPS-1            ILPSFLTRRIS------MAKIGIFLHTPFPSSEIFRTLW--CREDLLRGMLNADQVGFHLFEYARHFLTCCRRLLGLNYGMFPDSSGGHNL  224
T. pseudonana CCMP1335 TPS-2            LMPRMLREARN------ETKMVFFIHVPFPTSQVFRELE--HGEALLEGMLHADVVGFHAFDHARHFLNAAKRILGLGYES----LMGGLI  234
T. pseudonana CCMP1335 TPS-3            LLPRMLRNEEQ-MIQRPLTKTIFFLHIPFPVSMIFKEME--CGAAVLEGMLHADVVGFHGFTDARHFLSSAKRILGLSHES----LAGGLI  262
S. japonica TPS                         LLPQASRSRLS------GVKIGFFLHIPWPSSEVYRVLP--WRNELLKGMLSATLLGFHLFDYARHFLSACVRLLNLEHEA-----NRGSL  311
S. japonica TPS-2                       LLPSFLTRVLR------TARVGLFLHTPFPSSEIFRTLP--FREDLLRGMLNADQIGFHLFEYARHFLTCCKRILGLNEG---DSNNRGNT  407
S. henslowianum TPS                     LLPQASRSRLS------GVKIGFFLHIPWPSSEVYRVLP--WRNELLKGMLSATLLGFHLFDYARHFLSACVRLLNLEHEA-----NRGSL  311
U. pinnatifida TPS                      LLPQASRSRLS------GVKIGFFLHIPWPSSEVYRVLP--WRNELLKGMLSATLLGFHLFDYARHFLSACVRLLNLEHEA-----NRGSL  311
C. albicans TPS                         LLPEMLRQEIGNKK--KNIKIGFFLHTPFPSSEIYRILP--VRKEILEGVLSCDLIGFHTYDYARHFISSVSRIVPNVSTLPNGIKYQ---  231
C. higginsianum TPS1                    LLPEMLRREEIGDSK--KNVKIGFFLHTPFPSSEIYRILP--VREALLTGVLDCDLIGFHTYDYARHFLSSCSRIL-GTPTTPNGVDYN---  238
S. cerevisiae TPS                       LVPEMLRVKIHEKQL-QNVKVGWFLHTPFPSSEIYRILP--VRQEILKGVLSCDLVGFHTYDYARHFLSSVQRVL-NVNTLPNGVEYQ---  244
F. cylindrus CCMP1102 TPS               LLPRYLCEDGS---ATKYCRKVFFLHIPFPTSQIFRELE--CGEDILQGMLHADVVGFHAFDHARHFLNANKRIMGLNYEN----LQGGLI  273
V. carteri f. nagariensis TPS-1         VLPSLLRKRFN------RIRCGLFLHSPFPSSEIFRTFP--KREELLRSLLNADVIGFHTFDYARHFLSCCTRMLGLEHET-----SRGSI  234
C. reinhardtii TPS-1                    LLPSLLKQAVH------KMKVGFFLHTPFPSSEIYRTLP--VREEVLRAVLAADLVGFHTYDYARHFISSCTRIL-GLEGTPEGVENN---  298
C. reinhardtii TPS-2                    VLPSLLRKRFN------RIRCGLFLHSPFPSSEIFRTFP--KREELLRSLLNADLIGFHTFDYARHFLSCCSRMLGLEHET-----SRGSI  277
C. reinhardtii TPS-4                    SLPRVLFQYMC------AQQP-LNCPSSLPLTTPSHSPP--SPRPPLNTLTLLLPTGFHTFDYARHFLSCCSRMLGLEHET-----SRGSI  234
O. tauri TPS-1                          LVPEMLRQDVE------TMKIGWFLHTPFPSSEIYRMLP--MREALLHGCLAADLVGFHTYDYARHFVSATSRIL-GLEATPDGVETQ---  371
O. tauri TPS-2                          LLPTFLRKRFN------AVKCGFFLHCPFPSSEIFRTTP--TRDLILRGLLNADFVGFHTFDYARHFLSCCTRLLGLNHRM-----ERGSL  280
A. thaliana TPS-3                       FLPQYLKEYNN------KIKVGWFLHSPFPSSEIYKTLP--SRSELLRSVLAADLISFHTYDFARHFVNTCTRIL-GVEGTHEGVVYQ---  217
A. thaliana TPS-4                       LLPQYLKEYNS------KMKVGWFLHTPFPSSEMYKTLP--SRSDLLRSVLTADLVGFHTYDFARHFLNACMCIL-GVEATSEGIVDQ---  231
A. thaliana TPS-5                       VLPTFLRKRFN------RVKLGFFLHSPFPSSEIYRTLP--VRNELLRALLNADLIGFHTFDYARHFLSCCSRMLGLSYQS-----KRGTI  288
A. thaliana TPS-8                       VLPTFLRKRFN------RIKLGFFLHSPFPSSEIYRTLP--VRDEILRGLLNCDLIGFHTFDYARHFLSCCSRMLGLDYES-----KRGHI  286
Z. mays TPS                             FLPKCLKDHDI------NMKVGWFLHTPFPSSEIYRTLP--SRLELLRSVLCADLVGFHTYDYARHFVSACTRIL-GLEGTPEGVEDQ---  342
N. tabacum TPS                          FLPKCLKEYNS------QMKVGWFLHTPFPSSEIHRTLP--SRSELLRAVLAADLVGFHTYDYARHFVSACTRIL-GLEGTPEGVEDQ---  319
O. sativa TPS                           ALPTFLRRRFN------RLRIGFFLHSPFPSSEIYRSLP--VREEILRTLLNCDLIGFHTFDYARHFLSCCSRMLGIEYQS-----KRGYI  310
Z. marina TPS                           VLPTLLRRRFI------RLRMGFFLHSPFPSSEIYRTLP--VREEILNSLLCSDLIGFHTFDYARHFLSCCSRMMGLEYQS-----KRGYI  296
P. patens TPS                           CLPQKLKELNP------HMKVGWFLHTPFPSSEIYRTLP--LRSELLKAVLTADLIGFHTYDYARHFVSACTRIL-GLEGTPEGVEDQ---  276
P. patens TPS-X1                        CLPQELKTLNP------RMKVGWFLHTPFPSSEIYRTLP--LRSELLKAVLTADLIGFHTYDYARHFVSACTRIL-GLEGTPEGVEDQ---  395
C. elegans TPS-1                        LCGQIMRSLES------SLDIGFFIHIPFQPPANFMTKYKTVADPIMRALLRFTKVGFQTSRDRDTFVKLVAKHIKRTKIEYDSRLDRYT-  608
H. sapiens TPS                          LLPRMIRERL------PEAIVITFWHIPWPNSEVYSICP--WRERILEGLLGSSIIGFHTQFHANNFAESVDRFL-ESRIERADAAIS---  315
F. chinensis TPS                        MAANSIRDRCDKLG--LPIKMAFFLHIPFPSWDIMRLFP--WDDELLQGILGCDSVGFHVEDYCLNFIDCCQRRL-GCRVDREQMLVEH--  251
C. floridanus TPS                       LAANWIRQAADEKD--LRLKLGFFLHIPFPPWDIFRLFP--WADEILQGMLGCDMVGFHIQDYCLNFVDCCQRCL-GCRVDHKNLLVEH--  259
B. antarctica TPS                       LCANYIRELAEEKN--IEFQLAFFLHIPFPPWDIFRLFP--WSDEILQGMLGCDMVGFHIRDYCLNFVDCCQRNL-GCRVDRKNLLVEH--  271
```

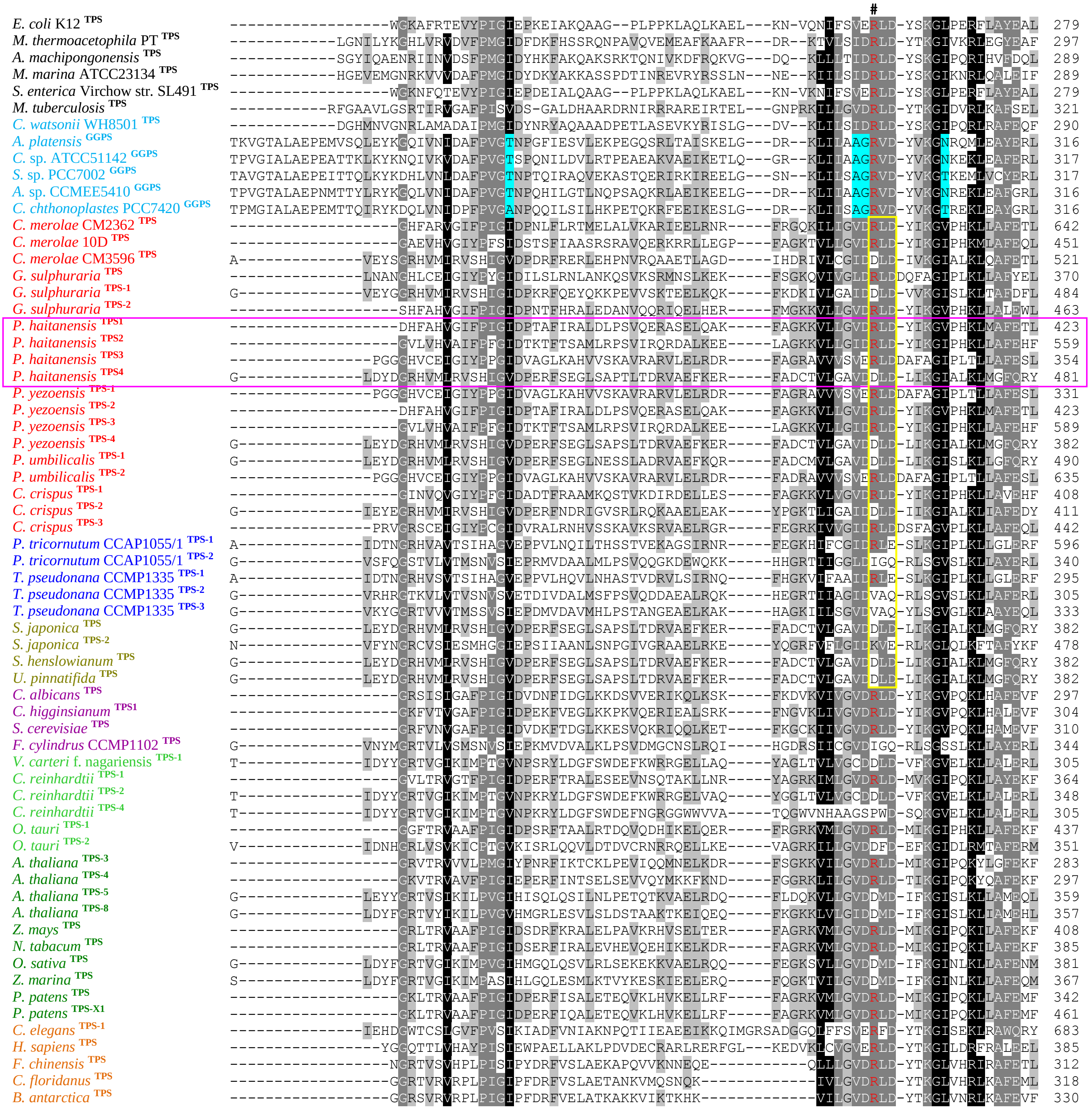
#
E. coli K12 TPS ------------------WGKAFRTEVYPIGIEPKEIAKQAAG--PLPPKLAQLKAEL---KN-VQNIFSVERLD-YSKGLPERFLAYEAL 279
M. thermoacetophila PT TPS ------------LGNILYKGHLVRVDVFPMGIDFDKFHSSRQNPAVQVEMEAFKAAFR---DR--KTVLSIDRLD-YTKGIVKRLEGYEAF 297
A. machipongonensis TPS ------------SGYIQAENRIINVDSFPMGIDYHKFAKQAKSRKTQNIVKDFRQKVG---DQ--KLLLTIDRLD-YSKGIPQRIHVFDQL 289
M. marina ATCC23134 TPS ------------HGEVEMGNRKVVVDAFPMGIDYDKYAKKASSPDTINREVRYRSSLN---NE--KIILSIDRLD-YSKGIKNRLQALEIF 289
S. enterica Virchow str. SL491 TPS ------------------WGKNFQTEVYPIGIEPDEIALQAAG--PLPPKLAQLKAEL---KN-VKNIFSVERLD-YSKGLPERFLAYEAL 279
M. tuberculosis TPS -----------RFGAAVLGSRTIRVGAFPISVDS-GALDHAARDRNIRRRAREIRTEL---GNPRKILLGVDRLD-YTKGIDVRLKAFSEL 321
C. watsonii WH8501 TPS ------------DGHMNVGNRLAMADAIPMGIDYNRYAQAAADPETLASEVKYRISLG---DV--KLILSIDRLD-YSKGIPQRLRAFEQF 290
A. platensis GGPS TKVGTALAEPEMVSQLEYKGQIVNIDAFPVGTNPGFIESVLEKPEGQSRLTAISKELG---DR--KLIIAAGRVD-YVKGNRQMLEAYERL 316
C. sp. ATCC51142 GGPS TPVGIALAEPEATTKLKYKNQIVKVDAFPVGTSPQNILDVLRTPEAEAKVAEIKETLQ---GR--KLIIAAGRVD-YVKGNKEKLEAFERL 317
S. sp. PCC7002 GGPS TAVGTALAEPEITTQLKYKDHLVNLDAFPVGTNPTQIRAQVEKASTQERIRKIREELG---SN--KLILSAGRVD-YVKGTKEMLVCYERL 317
A. sp. CCMEE5410 GGPS TPVGTALAEPNMTTYLRYKGQLVNIDAFPVGTNPQHILGTLNQPSAQKRLAEIKEELG---DR--KLIIAAGRVD-YVKGNREKLEAFGRL 316
C. chthonoplastes PCC7420 GGPS TPMGIALAEPEMTTQIRYKDQLVNIDPFPVGANPQQILSILHKPETQKRFEEIKESLG---DR--KLIISAGRVD-YVKGTREKLEAYGRL 316
C. merolae CM2362 TPS -------------------GHFARVGIFPIGIDPNLFLRTMELALVKARIEELRNR----FRGQKILIGVDRLD-YIKGVPHKLLAFETL 642
C. merolae 10D TPS -------------------GAEVHVGIYPFSIDSTSFIAASRSRAVQERKRRLLEGP----FAGKTVLLGVDRLD-YIKGIPHKMLAFEQL 451
C. merolae CM3596 TPS A--------------VEYSGRHVMIRVSHIGVDPDRFRERLEHPNVRQAAETLAGD-----IHDRIVLCGIDDLD-IVKGIALKLQAFETL 521
G. sulphuraria TPS ---------------LNANGHLCEIGIYPYGIDILSLRNLANKQSVKSRMNSLKEK-----FSGKQVIVGLDRLDDQFAGIPLKLLAFYEL 370
G. sulphuraria TPS-1 G--------------VEYGGRHVMIRVSHIGIDPKRFQEYQKKPEVVSKTEELKQK-----FKDKIVLGAIDDLD-VVKGISLKLTAFDFL 484
G. sulphuraria TPS-2 -------------------SHFAHVGIFPIGIDPNTFHRALEDANVQQRIQELHER----FMGKKVLIGVDRLD-YIKGVPHKLLALEWL 463
P. haitanensis TPS1 -------------------DHFAHVGIFPIGIDPTAFIRALDLPSVQERASELQAK----FAGKKVLLGVDRLD-YIKGVPHKLMAFETL 423
P. haitanensis TPS2 -------------------GVLVHVAIFPFGIDTKTFTSAMLRPSVIRQRDALKEE----LAGKKVLLGIDRLD-YIKGIPHKLLAFEHF 559
P. haitanensis TPS3 ----------------PGGGHVCEIGIYPPGIDVAGLKAHVVSKAVRARVLELRDR----FAGRAVVVSVERLDDAFAGIPLTLLAFESL 354
P. haitanensis TPS4 G--------------LDYDGRHVMLRVSHIGVDPERFSEGLSAPTLTDRVAEFKER----FADCTVLGAVDDLD-LIKGIALKLMGFQRY 481
P. yezoensis TPS-1 ----------------PGGGHVCEIGIYPPGIDVAGLKAHVVSKAVRARVLELRDR----FAGRAVVVSVERLDDAFAGIPLTLLAFESL 331
P. yezoensis TPS-2 -------------------DHFAHVGIFPIGIDPTAFIRALDLPSVQERASELQAK----FAGKKVLLGVDRLD-YIKGVPHKLMAFETL 423
P. yezoensis TPS-3 -------------------GVLVHVAIFPFGIDTKTFTSAMLRPSVIRQRDALKEE----LAGKKVLLGIDRLD-YIKGIPHKLLAFEHF 589
P. yezoensis TPS-4 G--------------LEYDGRHVMLRVSHIGVDPERFSEGLSAPSLTDRVAEFKER----FADCTVLGAVDDLD-LIKGIALKLMGFQRY 382
P. umbilicalis TPS-1 G--------------LEYDGRHVMLRVSHIGVDPERFSEGLNESSLADRVAEFKQR----FADCMVLGAVDDLD-LIKGISLKLLGFQRY 490
P. umbilicalis TPS-2 ----------------PGGGHVCEIGIYPPGIDVAGLKAHVVSKAVRARVLELRDR----FADRAVVVSVERLDDAFAGIPLTLLAFESL 635
C. crispus TPS-1 -------------------GINVQVGIYPFGIDADTFRAAMKQSTVKDIRDELLES----FAGKKVLVGVDRLD-YIKGIPHKLLAVEHF 408
C. crispus TPS-2 G--------------IEYEGRHVMIRVSHIGVDPERFNDRIGVSRLRQKAAELEAK----YPGKTLIGAIDDLD-IIKGIALKLIAFEDY 411
C. crispus TPS-3 ----------------PRVGRSCEIGIYPCGIDVRALRNHVSSKAVKSRVAELRGR----FEGRKIVVGIDRLDDSFAGVPLKLLAFEQL 442
P. tricornutum CCAP1055/1 TPS-1 A--------------IDTNGRHVAVTSIHAGVEPPVLNQILTHSSTVEKAGSIRNR----FEGKHIFCGIDRLE-SLKGIPLKLLGLERF 596
P. tricornutum CCAP1055/1 TPS-2 G--------------VSFQGSTVLVTMSNVSIEPRMVDAALMLPSVQQGKDEWQKK----HHGRTIIGGLDIGQ-RLSGVSLKLLAYERL 340
T. pseudonana CCMP1335 TPS-1 A--------------IDTNGRHVSVTSIHAGVEPPVLHQVLNHASTVDRVLSIRNQ----FHGKVIFAAIDRLE-SLKGIPLKLLGLERF 295
T. pseudonana CCMP1335 TPS-2 G--------------VRHRGTKVLVTVSNVSVETDIVDALMSFPSVQDDAEALRQK----HEGRTIIAGIDVAQ-RLSGVSLKLLAFERL 305
T. pseudonana CCMP1335 TPS-3 G--------------VKYGGRTVVVTMSSVSIEPDMVDAVMHLPSTANGEAELKAK----HAGKIILSGVDVAQ-YLSGVGLKLAAYEQL 333
S. japonica TPS G--------------LEYDGRHVMLRVSHIGVDPERFSEGLSAPSLTDRVAEFKER----FADCTVLGAVDDLD-LIKGIALKLMGFQRY 382
S. japonica TPS-2 N--------------VFYNGRCVSIESMHGGIEPSIIAANLSNPGIVGRAAELRKE----YQGRFVFLGIDKVE-RLKGLQLKFTAFYKF 478
S. henslowianum TPS G--------------LEYDGRHVMLRVSHIGVDPERFSEGLSAPSLTDRVAEFKER----FADCTVLGAVDDLD-LIKGIALKLMGFQRY 382
U. pinnatifida TPS G--------------LEYDGRHVMLRVSHIGVDPERFSEGLSAPSLTDRVAEFKER----FADCTVLGAVDDLD-LIKGIALKLMGFQRY 382
C. albicans TPS -------------------GRSISIGAFPIGIDVDNFIDGLKKDSVVERIKQLKSK----FKDVKVIVGVDRLD-YIKGVPQKLHAFEVF 297
C. higginsianum TPS1 -------------------GKFVTVGAFPIGIDPEKFVEGLKKPKVQERIEALSRK----FNGVKIIVGVDRLD-YIKGVPQKLHALEVF 304
S. cerevisiae TPS -------------------GRFVNVGAFPIGIDVDKFTDGLKKESVQKRIQQLKET----FKGCKIIVGVDRLD-YIKGVPQKLHAMEVF 310
F. cylindrus CCMP1102 TPS G--------------VNYMGRTVLVSMSNVSIEPKMVDVALKLPSVDMGCNSLRQI----HGDRSIICGVDIGQ-RLSGSSLKLLAYERL 344
V. carteri f. nagariensis TPS-1 T--------------IDYYGRTVGIKIMPTGVNPSRYLDGFSWDEFKWRRGELLAQ----YAGLTVLVGCDDLD-VFKGVELKLLALERL 305
C. reinhardtii TPS-1 -------------------GVLTRVGTFPIGIDPERFTRALESEEVNSQTAKLLNR----YAGRKIMLGVDRLD-MVKGIPQKLLAYEKF 364
C. reinhardtii TPS-2 T--------------IDYYGRTVGIKIMPTGVNPKRYLDGFSWDEFKWRRGELVAQ----YGGLTVLVGCDDLD-VFKGVELKLLALERL 348
C. reinhardtii TPS-4 T--------------IDYYGRTVGIKIMPTGVNPKRYLDGFSWDEFNGRRGGWWVVA----TQGWVNHAAGSPWD-SQKGVELKLLALERL 305
O. tauri TPS-1 -------------------GGFTRVAAFPIGIDPSRFTAALRTDQVQDHIKELQER----FRGRKVMLGVDRLD-MIKGIPHKLLAFEKF 437
O. tauri TPS-2 V--------------IDNHGRLVSVKICPTGVKISRLQQVLDTDVCRNRRQELLKE----VAGRKILLGVDDFD-EFKGIDLRMTAFERM 351
A. thaliana TPS-3 -------------------GRVTRVVVLPMGIYPNRFIKTCKLPEVIQQMNELKDR----FSGKKVILGVDRLD-MIKGIPQKYLGFEKF 283
A. thaliana TPS-4 -------------------GKVTRVAVFPIGIEPERFINTSELSEVVQYMKKFKND----FGGRKLILGVDRLD-TIKGIPQKYQAFEKF 297
A. thaliana TPS-5 G--------------LEYYGRTVSIKILPVGIHISQLQSILNLPETQTKVAELRDQ----FLDQKVLLGVDDMD-IFKGISLKLLAMEQL 359
A. thaliana TPS-8 G--------------LDYFGRTVYIKILPVGVHMGRLESVLSLDSTAAKTKEIQEQ----FKGKKLVLGIDDMD-IFKGISLKLIAMEHL 357
Z. mays TPS -------------------GRLTRVAAFPIGIDSDRFKRALELPAVKRHVSELTER----FAGRKVMLGVDRLD-MIKGIPQKILAFEKF 408
N. tabacum TPS -------------------GRLTRVAAFPIGIDSERFIRALEVHEVQEHIKELKDR----FAGRKVMLGVDRLD-MIKGIPQKILAFEKF 385
O. sativa TPS G--------------LDYFGRTVGIKIMPVGIHMGQLQSVLRLSEKEKKVAELRQQ----FEGKSVLLGVDDMD-IFKGINLKLLAFENM 381
Z. marina TPS S--------------LDYFGRTVGIKIMPASIHLGQLESMLKTVYKESKIEELERQ----FQGKTVILGVDDMD-IFKGINLKLLAFEQM 367
P. patens TPS -------------------GKLTRVAAFPIGIDPERFISALETEQVKLHVKELLRF----FAGRKVMLGVDRLD-MIKGIPQKLLAFEMF 342
P. patens TPS-X1 -------------------GKLTRVAAFPIGIDPERFIQALETEQVKLHVKELLRF----FAGRKVMLGVDRLD-MIKGIPQKLLAFEMF 461
C. elegans TPS-1 ---------------IEHDGWTCSLGVFPVSIKIADFVNIAKNPQTIIEAEEIKKQIMGRSADGGQLFFSVERFD-YTKGISEKLRAWQRY 683
H. sapiens TPS -----------------YGGQTTLVHAYPISIEWPAELLAKLPDVDECRARLRERFGL---KEDVKLCVGVERLD-YTKGILDRFRALEEL 385
F. chinensis TPS ------------------NGRTVSVHPLPISIPYDRFVSLAEKAPQVVKNNEQE-----------QLLGVDRLD-YTKGLVHRIRAFETL 312
C. floridanus TPS ------------------GGRTVRVRPLPIGIPFDRFVSLAETANKVMQSNQK-------------IVLGVDRLD-YTKGLVHRLKAFEML 318
B. antarctica TPS ------------------GGRSVRVRPLPIGIPFDRFVELATKAKKVIKTKHK-------------VILGVDRLD-YTKGLVNRLKAFEVF 330

```
                                                  *
E. coli K12 TPS                           LEKYP-QHHGKIRYTQIAPTSRG----------DVQAYQDIRHQLENEAGRINGKYGQL---GWTPLYYLNQHFDRKLLMKIFRYSDVGLV  356
M. thermoacetophila PT TPS                LKKNP-SWHGRVILVLVVVPSRV----------RVEHYQQMKRQIDEKVGDINGKFGKV---DWTPILYTYRYLPEPQLQALYNLCDTALI  374
A. machipongonensis TPS                   LQQHK-ELRGKVSMIMVVVPSRD----------RVQSYKELKEEIDLLVGRINSEYSTL---DWVPVHYFYRSFPFEELSAFYSMSDVALV  366
M. marina ATCC23134 TPS                   FEEYP-EYLEKMVLFMVVVPSRD----------TVPRYQALKTEIDELVGRINGKYGRV---SWTPVHYFYRSFPLEALSAFYRMANVALV  366
S. enterica Virchow str. SL491 TPS        LENYP-QHRGKIRYTQIAPTSRG----------EVQAYQDIRHQLETEAGRINGKYGQL---GWTPLYYLNQHFDRKLLMKIFRYSDVGLV  356
M. tuberculosis TPS                       LAEGR-VKRDDTVVVQLATPSRE----------RVESYQTLRNDIERQVGHINGEYGEV---GHPVVHYLHRPAPRDELIAFFVASDVMLV  398
C. watsonii WH8501 TPS                    IEENE-EFREEVSLLILVVPSRD----------QVPMYANLKKEIELLVGSINGKFGTI---NWRPIHYFYRSYPLHSLSAFYRMSHVALV  367
A. platensis GGPS                         LERRQ-DLHGKVQFLMTCVSAAD----------GMRVYKTAQHQIEQLVGKINGRFARL---DWMPTLLFTQPIPLSDLFSYYRLADVCWT  393
C. sp. ATCC51142 GGPS                      LERRP-ELHGKVNFIMTCVQAAT----------GMQVYQEAQREIEYLVGKINGRFAKF---DWIPIRLSTQPIPLLDLFCYYRAADICWT  394
S. sp. PCC7002 GGPS                       LERRP-ELQTKVNLVVAAAKAAS----------GMRVYKNAQSEIERLVGRINGRFAKL---NWTPILLFTSALSYEELLGFFGAADIAWI  394
A. sp. CCMEE5410 GGPS                      LERHP-DLHGKINFVMTCVTPAT----------GMRVYKTAQSQIEQLVGKINGRFAKL---GWTPILLYTQPLSLDDLLCYYRTADICWT  393
C. chthonoplastes PCC7420 GGPS            LARRP-DLHGKINMLVTCVASAS----------GMRVYKTAQTQIEQLAGKINGRYGNL---DWLPIMLYTQPVPLPDLLCYYKAADICWT  393
C. merolae CM2362 TPS                     LKKYP-EWHEKVVLAQIAVPSRI----------EVEEYRKLIAYTNELVGRINGRFGSV---EYAPIMFINQSIPFEELCALYHVADVAVI  719
C. merolae 10D TPS                        LESHP-EWIGRAVLLQVVVPSAPDLSLDAVDERASSDYLALRAEVEELAGRINGRFGTV---EDLPIMVRIMEPDLEDLVVLYSIADVAVI  538
C. merolae CM3596 TPS                     LQTYP-GYHRRLVLMQAAIPKAA----------RVK--PYVRDEIRSLVERINGRYG---SAEYRPVVYIERDITFDERVAMYSLADAIFL  596
G. sulphuraria TPS                        LKRNR-KWQQKVVFVEIAVASER--------PSQ--TCLKQAEHVYELVGLINSEFGTF---GFCPLHFVNRELSPEDVCALLSIGDICIV  447
G. sulphuraria TPS-1                      LANYP-SYRNRIVLNQVAIPKTS----------RVK--ENVRNEIRSLVDQINERYG---SQSYLPVMYFEREISFDERVAMYSRADAFFL  559
G. sulphuraria TPS-2                      LTKYS-EWMHKVVLVQIAVPTRT----------EIEEYKKLSSQTNELVGRINGKFGTV---DHSPIMFINQSLAFEELVALYTVADVAIV  540
P. haitanensis TPS1                       LARHP-EWNERAVLVQIAVPSRT----------EVEEYKKLSSQTHELVGRINGKFGSV---DYSPIVFINQSVNFHDLVALYSVADVCVV  500
P. haitanensis TPS2                       LETYP-EYVGHVVLVQIATPSQT----------TSEEYAGFRAEILEEVGRINGRFGTV---DDMPIHYREHAMSFDTLCALYSMADVAVI  636
P. haitanensis TPS3                       LHKHP-EYVTAVVLVLVATIPRH--------PRQLSSYRALASQINTSVGRINSTYGSI---GTSPVHFINAELPQDELYALMSVGSVCVV  433
P. haitanensis TPS4                       LDTAP-KMRGKVVLVQVAIPKAA----------RVK--AAVRNEIRELVAAINDKHGD--GSGRRPVWYLEESISFESRLALYSIMDALVV  557
P. yezoensis TPS-1                        LHKHP-EYVTSVVLVLVATIPRH--------PRQLSSYRALASQINTSVGRINSTYGSI---GTSPVHFINAELPQDELYALMSVGSVCVV  410
P. yezoensis TPS-2                        LARHP-EWNERAVLVQIAVPSRT----------EVEEYKKLSSQTHELVGRINGKFGSV---DYSPIVFINQSVNFHDLVALYSVADVCVV  500
P. yezoensis TPS-3                        LETYP-EYVGHVVLVQIATPSQT----------TSEEYAGFRAEILEEVGRINGRFGTV---DDMPIHYREHAMSFDTLCALYSMADVAVI  666
P. yezoensis TPS-4                        LDSAP-KMRGKVVLVQVAIPKAA----------RVK--ESVRNEIRELVAAINNKHGD--GSGRRPVWYLEESISFESRLALYSIMDALVV  458
P. umbilicalis TPS-1                      LDTAP-NMRGKVVLVQVAIPKAA----------RVK--ESVRNEIRELVAAINDKHGD--GSGRRPVWYLEESISFESRLALYSIMDALVL  566
P. umbilicalis TPS-2                      LHKYP-EYVTSAVLVLVATIPRH--------PRQLSSYRALASQINTSVGRINSTYGSI---GSSPVHFINAELPQDELYALLSVGSVCVV  714
C. crispus TPS-1                          LECHP-EWIGRVVMLQVTTPSMS----------ASEEYHAFRSEILEMVGRINGRFATL---EDMPIHYREVTMTFEQMCALYSVADVAII  485
C. crispus TPS-2                          LSTSSERVRSNVALIQVAIPKAA----------RVN--VSVRQEIRELVDKINSTYG---TADHKPVLYIEQNITFDERMALYSVCDALLL  487
C. crispus TPS-3                          LTDHP-EMVGKVVLIQAAMLPKQ--------GKSRDAQHTQQNQINELVGRINSSFGTF---AFSPVHYINTELSPTEIHALMCIGHACVV  521
P. tricornutum CCAP1055/1 TPS-1           LRRCP-EWVGKIVLVQVGISAFE----------RGDDYSITKHEVVKLVDKINQEWPGT----IQFQECYESEMRLQQRMALLRAADVVVV  672
P. tricornutum CCAP1055/1 TPS-2           LQDYP-SWVNSVVMIQLVIVPGS----------RKADEAVTTSELRSLVKRIQDKFGP---EVIDYVELAGSSIPMDRRLAMWKASDILMS  417
T. pseudonana CCMP1335 TPS-1              LQRCP-EWAGKIVVVQVGISAFE----------RGDDYSKTRNEVLSMVTNINNRWPGT----VQFQECAESEMRLQQRMALLRAADIVMV  371
T. pseudonana CCMP1335 TPS-2              LTDYP-VWQSKVVLLQRCLIPGN----------RRVDEADTLREVRFLVKKIEMKFGAE-GENIEWIVLG-SVMPIDQRLSIWRSSQVALH  383
T. pseudonana CCMP1335 TPS-3              LKDIP-SGRDKLVLVQRCLITGA----------RRLDEARTIREIRLIVKRIQKKFGE---QVIDYQEFFGSALPIDQRLALWKASDILMC  410
S. japonica TPS                           LDSAP-KMRGKVVLVQVAIPKAA----------RVK--ESVRNEIRELVAAINNKHGD--GSGRRPVWYLEESISFESRLALYSIMDALVV  458
S. japonica TPS-2                         LLERP-HLADKIVLLQVGISALE----------REQDYHKCLNELRTLAQKINSECAPSP--DRPVLVLQLEDEHSFQRLPYLCLADCLIN  556
S. henslowianum TPS                       LDSAP-KMRGKVVLVQVAIPKAA----------RVK--ESVRNEIRELVAAINNKHGD--GSGRRPVWYLEESISFESRLALYSIMDALVV  458
U. pinnatifida TPS                        LDSAP-KIRGKVVLVQVAIPKAA----------RVK--ESVRNEIRELVAAINNKHGD--GSGRRPVWYLEESISFESRLALYSIMDALVV  458
C. albicans TPS                           LNENP-EWIGKVVLVQVAVPSRG----------DVEEYQSLRSTVSELVGRINGEFGTV---EFVPIHYLHKSIPFDELISLYNISDVCLV  374
C. higginsianum TPS1                      LTEHP-EWIGKIVLVQVAVPSRQ----------DVEEYQNLRAVVNELVGRINGKFGTI---EFMPIHFLHQSVSFDELTALYAVSDVCLV  381
S. cerevisiae TPS                         LNEHP-EWRGKVVLVQVAVPSRG----------DVEEYQYLRSVVNELVGRINGQFGTV---EFVPIHFMHKSIPFEELISLYAVSDVCLV  387
F. cylindrus CCMP1102 TPS                 LQDYP-SWQAKVVMVLRILLPGS----------RKRDEQITVRELRQIVLRIQEKYGS---AVIDYKEITGSNLPLDQRLALWKASDVLML  421
V. carteri f. nagariensis TPS-1           LEQHS-EWRGQLVLVQITNPPRS----------TGRDITELHRCVNNLVDSINRKYG---KGSYQPVQYLERHVPLHERMAFYSVADCAVV  382
C. reinhardtii TPS-1                      LEEHP-EWRDKVLLVQIAVPSRT----------DVPEYQKLRSMVHEIVGRINGQYGTL---TYVPIYHLDTSLSFTELCALYAVTDVALV  441
C. reinhardtii TPS-2                      LDTHP-EWRGQLVLVQITNPPRS----------TGRDIAELHRCVLGLVDSINRKYG---KGSYQPVQYLERHVPLHERMAFYSIADCAVV  425
C. reinhardtii TPS-4                      LDTHP-EWRGQLVLVQITNPPRS----------TGRDIAELHRCVLGLVDSINRKYG---KGSYQPVQYLERHVPLHERMAFYSIADCAVV  382
O. tauri TPS-1                            LIENP-EWKDKCCLIQIAVPTRS----------EVPEYQKLASTVHEIVGRINGRFGSI---GSIPIQHLDCSMQFPELCALYSVTDVMLV  514
O. tauri TPS-2                            LEEHP-RLAEQVCLMQTCTPPRS----------SGRDINELRNTVNELVERINAKYS---VNGVPVLKFEIKPVAIHERIALHSVADVSVV  428
A. thaliana TPS-3                         LDENP-NWRDKIVLVQIAVPTRN----------EVPEYQKLKNQVHRLVGRINGRFGSV---SSLPIHHMDCSVDSNYLCALYAISDVMLV  360
A. thaliana TPS-4                         LEENA-EWRGKVMLLQIAVPTRN----------GIGEYQKIKDQCHYHVGRINGRFGSI---SSVPIIHLDCSIDFNQLCALYAITDVLLV  374
A. thaliana TPS-5                         LTQHP-EKRGRVVLVQIANPARG----------RGKDVQEVQSETEATVKRINEMFG---RPGYQPVVLIDTPLQFFERIAYYVIAECCLV  436
A. thaliana TPS-8                         FETYW-HLKGKVVLVQIVNPARS----------SGKDVEEAKRETYETARRINERYG---TSDYKPIVLIDRLVPRSEKTAYYAAADCCLV  434
Z. mays TPS                               LEENP-DWNNKVVLLQIAVPTRT----------DVPEYQKLTSQVHEIVGRINGRFGTL---TAVPIHHLDRSLDFHALCALYAVTDVALV  485
N. tabacum TPS                            LEENP-YWRDKVVLLQIAVPTRT----------DVPEYQKLTSQVHEIVGRINGRFGTL---TAVPIHHLDRSLDFHALCALYAVTDVALV  462
O. sativa TPS                             LRTHP-KWKGRAVLVQIANPARG----------KGKDLEAVQAEIRESCDRINKEFG---QSGYSPVIFIDQSVPSAVRLAYYTVAECVVV  458
Z. marina TPS                             LKLRP-NWQGRAVLVQIANPARG----------RGKGLESVEVEIRDICERINQQFG---RVGYKPVVYINRSVSLKERIAYYTIAECVVV  444
P. patens TPS                             LEKNP-QWREKVMLVQIAVPTRT----------DVHEYQRLTSQVHEIVGRINGRFGTV---TFVPINHLDRSLAFHELCALYASTDVVLV  419
P. patens TPS-X1                           LEKNP-EWREKVMLVQIAVPTRT----------DVHEYQRLTSQVHEIVGRINGRYGTV---TFVPIHHLDRSLAFHELCALYASTDVALV  538
C. elegans TPS-1                          FEKYP-DRIGKDVLFQVAVTNRR----------SVDSYRQYQDDVLAVADLINQKFKSEDYPEWKPVIFETDGLPRTRLIAHYLAMDIGVV  763
H. sapiens TPS                            FTRHP-EWVGKLVLLQIAAPSRG----------TLPAYRQLHDECRRYVEELNQRYGSE---DYSPVLMVDKHHPQEQVYEFYRAADICMV  462
F. chinensis TPS                          LQKHP-EHIEHVTFLQVAVPSRT----------DVKEYQELKEDLDQLIGRINGRFSTP---NWSPIRYIYGCVSQEQLAAFYRDSSVAVV  389
C. floridanus TPS                         LEKHP-EHREQVTMLQIAVPSRT----------DVREYQDLKLEMDQLIGCINGRFTTP---NWSPIRYIYGCVSQDELAAFYRDAAVALV  395
B. antarctica TPS                         LERHP-EHIGTVSFLQIAVPSRT----------DVLEYQQLKEEMDQLVGRINGRFSTA---IWSPIRYIFGCVGQSELAAYYRDAAVALV  407
```

	# ### #	
E. coli K12 TPS	TPLRDGMNLVAKEYVAAQDPANP-------------------GVLVLSQFAGAANEL-TSALIVNP-----------YDRDEVAAALDRAL	416
M. thermoacetophila PT TPS	TPLRDGMNLVAKEYIASKVDCR--------------------GVLILSEMAGASKEA-VEAILINP-----------YNIEEIADAILKAL	433
A. machipongonensis TPS	TPLRDGMNLVCKEFVASKTNQT--------------------GVLILSEMAGASKEL-QDAILVNP-----------NDRQGVVDAIYEGL	425
M. marina ATCC23134 TPS	TPLRDGMNLVCKEYIASRLDQT--------------------GVLILSEMAGSSKEL-SDAILINP-----------NDIKQIVAALHQAL	425
S. enterica Virchow str. SL491 TPS	TPLRDGMNLVAKEFVAAQDPANP-------------------GVLVLSQFAGAANEL-TSALIVNP-----------YDRDDVAAALNRAL	416
M. tuberculosis TPS	TPLRDGMNLVAKEYVACRSDLG--------------------GALVLSEFTGAAAEL-RHAYLVNP-----------HDLEGVKDGIEEAL	457
C. watsonii WH8501 TPS	TPLRDGMNLVCKEFVASKLDKK--------------------GVLILSETAGSAKEL-SDAILINP-----------NDTNQMVEAMKEAL	426
A. platensis GGPS	TPLRDGLNLVAKEYVVAREDRG--------------------GVLVLSEFVGAAVEL-PQAVLTNP-----------YSTQRMDEAIEFAL	452
C. sp. ATCC51142 GGPS	TPLRDGLNLVAKEYIVAHEGKD--------------------GVLVLSEFVGAATEL-PQAILTNP-----------YSIKLMDEAIDQAL	453
S. sp. PCC7002 GGPS	TPLRDGLNLVAKEYVVAHGCDD--------------------GVLILSEFAGSAVEL-PDAILTNP-----------YAAKRMDESIDQAL	453
A. sp. CCMEE5410 GGPS	TPLRDGLNLVAKEYIIAKDKQP--------------------GVLVLSEFVGAAVEL-PEAVLTNP-----------YSMDRMDEAIDKAL	452
C. chthonoplastes PCC7420 GGPS	TPLRDGLNLVAKEYIITHEKQE--------------------GVLVLSEFVGAAVEL-PDAILTNP-----------YSIDRMDESIEQAL	452
C. merolae CM2362 TPS	TSIRDGMNIVSYEYVMCQREK--------------------CGVLILSEFAGSAQSL-SGAIRVNP-----------WNIEELAAAMHEAL	778
C. merolae 10D TPS	TSLREGMGIMSYEFAVCQGE--------------------KRGVLVMSEFCGAAHSL-PGAVLCNP-----------WSIDEVAETLHRAM	597
C. merolae CM3596 TPS	TPIRDGLNIVPYEYVVS--------------------ASKSKGQLILSEFTGCSRAL-SGAVRINP-----------WNREEVAAAIDSVL	655
G. sulphuraria TPS	SSTRDGISIVSYEWVLCQHSG-------------------NHGVLVLSEFSGSALSF-SSAQHVNP-----------WNTDELVSVIENSL	507
G. sulphuraria TPS-1	TPIRDGLNIVPYEYIVS--------------------ASEGKGQLILSEFTGCSRAL-SGAVRVNP-----------WNIEEVASVIDRVM	618
G. sulphuraria TPS-2	SSVRDGMNIVSYEYVMCQQAN--------------------KGVLILSEFAGSAQSL-SGAIRVNP-----------WNVEELANAIHEAL	599
P. haitanensis TPS1	SSIRDGMNIVSYEYVMCQRER--------------------HGVLVLSEFAGSAQSL-SGALRVNP-----------WNIEELATALHDAL	559
P. haitanensis TPS2	TSLRDGMNIVSYEYIVCQAK--------------------NRGVLVLSEYTGAAQSL-PGALLCNP-----------WSVEEVSHTLHVAL	695
P. haitanensis TPS3	SSIRDGMSIVPYEWAICQHAG-------------------NRGPLILSEFAAAAHSF-STARHVNP-----------WDVDDLRDKLAACL	493
P. haitanensis TPS4	TPIRDGLNIIPYEYIVS--------------------TSEGKGQLVLSEFTGCSRAL-SSAVRVNP-----------WDLEKLCGVLDMVV	616
P. yezoensis TPS-1	SSIRDGMSIVPYEWAICQHAG-------------------NRGPLILSEFAAAAHSF-STARHVNP-----------WDVDDLRDKLAACL	470
P. yezoensis TPS-2	SSIRDGMNIVSYEYVMCQRER--------------------HGVLVLSEFAGSAQSL-SGALRVNP-----------WNIEELATALHDAL	559
P. yezoensis TPS-3	TSLRDGMNIVSYEYIVCQAK--------------------NRGVLVLSEYTGAAQSL-PGALLCNP-----------WSVEEVSHTLHVAL	725
P. yezoensis TPS-4	TPIRDGLNIIPYEYIVS--------------------TSEGKGQLILSEFTGCSRAL-SSAERVNP-----------WDLEKLSNVLDMVV	517
P. umbilicalis TPS-1	TPIRDGLNIIPYEYIVS--------------------TSEGKGQLVLSEFTGCSRAL-SSAVRVNP-----------WDIEELRGVLDMVV	625
P. umbilicalis TPS-2	SSIRDGMSIVPYEWAICQHAG-------------------NRGPLILSEFAAAAHSF-STARHVNP-----------WDVDDLRDKLAACL	774
C. crispus TPS-1	TSLRDGMNIVSHEYIMCQKE--------------------QNGVLILSEFTGAAQNL-PGALLVNP-----------WNVEMVSDAIFQSL	544
C. crispus TPS-2	TPIRDGLNIIPYEYIVS--------------------TPQGKGQLILSEFTGCSRAL-SSAVRVNP-----------WNVDELRDAIDNVI	546
C. crispus TPS-3	STVRDGMGIVPHEWTVCQHGG-------------------NNGPIVLSEFSGAAQSL-ATALHVNP-----------WNIGEMAVKIKIAL	581
P. tricornutum CCAP1055/1 TPS-1	TSIRDGLNILPLEFTTAHQDAMTEMGRKDG---------RKRGLCILSEFTSCTRVM-RGALHVNP-----------WKISEIANAFNTVL	742
P. tricornutum CCAP1055/1 TPS-2	TPIREGLNHWPMEYIHAHKEPEVP------------------GVVITSEFSAISSIL-NGALRVNP-----------FDIQMTVSTIDKAL	478
T. pseudonana CCMP1335 TPS-1	TTIRDGLNIIPLEFTIAHLDALSEQGRNDG---------RKRGLCILSEFSSCTRVM-RGALHVNP-----------WKISEIATAFFQAL	441
T. pseudonana CCMP1335 TPS-2	TPIREGLNIGPLEYVYARKEPSDP------------------GVVIASEFSAVSSVL-NGALRVNP-----------FDVQMCVTSIDCAL	444
T. pseudonana CCMP1335 TPS-3	TDVRGGLNIWPLEFVYAQKYSDRP------------------GIVIASEFTAVFGIL-NGALRISP-----------FDMKLTLGTIDKAI	471
S. japonica TPS	TPIRDGLNIIPYEYIVS--------------------TSEGKGQLILSEFTGCSRAL-SSAERVNP-----------WDLEKLSNVLDMVV	517
S. japonica TPS-2	TAVRDGLNNLPLEYVFVHESARKN----------------NPGVMMLSEFTSCMRVM-RGAIRINP-----------WKVEEVADVLAEVA	639
S. henslowianum TPS	TPIRDGLNIIPYEYIVS--------------------TSEGKDQLILSEFTGCSRAL-SSAERVNP-----------WDLEKLSNVLDMVV	517
U. pinnatifida TPS	TPIRDGLNIIPYEYIVS--------------------TSEGKGQLILSEFTGCSRAL-SSAERVNP-----------WDLEKLSNVLDMVV	517
C. albicans TPS	SSTRDGMNLVSYEYIACQQDR--------------------KGVLILSEFAGAAQSL-NGALIVNP-----------WNTEDLSEAIKESL	433
C. higginsianum TPS1	SSTRDGMNLVSYEYIATQRER--------------------HGVMILSEFTGAAQSL-NGSLIVNP-----------WNTEELANAIHDAV	440
S. cerevisiae TPS	SSTRDGMNLVSYEYIACQEEK--------------------KGSLILSEFTGAAQSL-NGAIIVNP-----------WNTDDLSDAINEAL	446
F. cylindrus CCMP1102 TPS	TPIREGLNHWPLEYIYCKRD-MAP------------------GVVIASEFSAVCSIL-NGALRVNP-----------FDIQMTVTIMDKAL	481
V. carteri f. nagariensis TPS-1	TATRDGMNLVPYEYVVCRQGPDGWDG-------SGGSGGRRESMLVVSEFVGCSPSL-SGAIRVNP-----------WSVESTADGIYAAI	454
C. reinhardtii TPS-1	TSLRDGMNLVSYEYVACQS-DN-------------------AGVLVLSEFAGAAQSLGAGALLVNP-----------WNISDMATAIYDAL	501
C. reinhardtii TPS-2	TATRDGMNLVPYEYVVCRQGPDGWDGGAAAGGAPGGGGSKRESMLVVSEFVGCSPSL-SGAIRVNP-----------WSVESTADGIYAAI	504
C. reinhardtii TPS-4	TATRDGMNLVPYEYVVCRQGPDGWDGGAAAGGAPGGGGSKRESMLVVSEFVGCSPSL-SGAIRVNP-----------WSVD----------	451
O. tauri TPS-1	TSQRDGMNLVSYEFVSCQNKSN-------------------AGVLVLSEFAGAAQSIGAGALLVNP-----------HNVTEVAHAIKEAL	575
O. tauri TPS-2	TATRDGMNLAPYEYITCRQGPSDEARG-----TAEGFSLPRQSALIISEFTGCSPSL-SGALRVNP-----------WHIQDVADAMYKAL	502
A. thaliana TPS-3	TSLRDGLNLVSHEFVACQEAK--------------------RGVLILSEFAGAGQSLGAGALLVNP-----------WNVTEVSSAIKKAL	420
A. thaliana TPS-4	TSLRDGMNLVSSEFIACQKAE--------------------KGVLILSEFAGAGQSLGAGAILVNP-----------WNIKEVSSAIGEAL	434
A. thaliana TPS-5	TAVRDGMNLIPYEYIICRQGNPKLNETIGL-----DPSAAKKSMLVVSEFIGCSPSL-SGAIRVNP-----------WNIDAVTEAMDYAL	510
A. thaliana TPS-8	NAVRDGMNLVPYKYIVCRQGTR---SNKAV-----VDSSPRTSTLVVSEFIGCSPSL-SGAIRVNP-----------WDVDAVAEAVNSAL	505
Z. mays TPS	TSLRDGMNLVSYEYVACQGSK--------------------KGVLILSEFAGAAQSLGAGAILVNP-----------WNITEVADSIRHAL	545
N. tabacum TPS	TSLRDGMNLVSYEFVACQESK--------------------KGVLILSEFAGAAQSLGAGAILVNP-----------WNITEVAAXIGQAL	522
O. sativa TPS	TAVRDGMNLTPYEYIVCREGIP--GSECAP-----EVSGPKKSMLVVSEFIGCSPSL-SGAIRVNP-----------WNIEATAEALNEAI	530
Z. marina TPS	SAVRDGMNLIPYEYTVCKQGIA--EPESDS-----LFADPKKSMLVVSEFIGCSPSL-SGAIKINP-----------WNSEATAEAMSDAI	516
P. patens TPS	TSLRDGMNLVSYEYVACQHMKN------------------TEGVLVLSEFAGAAQSLGAGAILVNP-----------WNIREMYKAIEEAL	481
P. patens TPS-X1	TSLRDGMNLVSYEYVACQNFKN------------------TEGVLVLSEFAGAAQSLGAGAILVNP-----------WNIREMYIAIEEAL	600
C. elegans TPS-1	TPSKDGMNLVAKEMLVCNPTAS----------------------LVLSTGAGTEVQLSNAQFYSEQEGKCYHRVEEISNTEAFADNFFAAA	832
H. sapiens TPS	TSLHDGMNLVAKEFVAARDDEQ--------------------GVLLLSTFAGASREL-LEALIVNP-----------YDTAMTSEALLQAL	521
F. chinensis TPS	TPLRDGMNLVAKEFVACQVSEP--------------------GVLMLSPFAGAGSSM-HEALLVNP-----------YETDEFAEVMHRAL	448
C. floridanus TPS	TPLRDGMNLVAKEFVACQINQPP-------------------GVLIVSPFAGAGEMM-HEALICNP-----------YEIDEAAEVIHRAL	455
B. antarctica TPS	TPLRDGMNLVAKEFVACQINEPP-------------------GVLIVSPFAGAGETM-HEALLCNP-----------YELDAAAEVINRAL	467

```
E. coli K12 TPS                       TMS---LAERISRHAEMLDVIVKNDINHWQECFISDLKQIVPR------------------------------------------------  456
M. thermoacetophila PT TPS            EMP---EDEQIWRCRMIQSRLKRNDIFKWASSFVRSLGAAAEEGK----------------------------------------------  475
A. machipongonensis TPS               SMK---ESEQKLRIKAMQESLKKYDVFQWVKVFMDRVEHVKEKQAELTSKDVD--------------------------------------  475
M. marina ATCC23134 TPS               TMP---KDEQKFHMEIMQNSLKRYNIHHWVKLFLDRLTEVKEQQSYMHTKILE--------------------------------------  475
S. enterica Virchow str. SL491 TPS    TMP---LAERISRHAEMLDVIVKNDINRWQERFIHDLKEVTPRSPERQQQNNVATFPKLA-------------------------------  473
M. tuberculosis TPS                   NQT---EEAGRRRMRSLRRQVLAHDVDRWAQSFLDALAGAHPRGQG---------------------------------------------  500
C. watsonii WH8501 TPS                KMP---EEEQIARMETMQKSLKRYDINAWVKLFMKGLEQVKEEQENLRTKPI---------------------------------------  475
A. platensis GGPS                     AMD---PEEQKRRMAEMRKTVVKYDVKYWADHVFERFESMTHQKAAVKTTVAV--------------------------------------  502
C. sp. ATCC51142 GGPS                 DMS---PEEQQEKMAKMYETVTKYDVKYWSDRLLNYFAKMTREFVDDKEPALN--------------------------------------  503
S. sp. PCC7002 GGPS                   AMP---VEEQQRRMKSMYQAIQRYDVQQWANHMFREAKATAVLGKEPTPV-----------------------------------------  500
A. sp. CCMEE5410 GGPS                 AMS---PEESQAAMTKMFETVTKYDVDYWANHLLQKFKAIQSAPSNQASVS----------------------------------------  500
C. chthonoplastes PCC7420 GGPS        AMS---PEEQKERMAKMYDIVTKYDIQCWADHLFEMFEKTKHQTAKQQQAVAV--------------------------------------  502
C. merolae CM2362 TPS                 TMS---DREREMKHWKLYRYVTTHTASYWAQSFVS-ELQQLRSTQQQQQQP----------------------------------------  825
C. merolae 10D TPS                    ALS---KMERELRYQKLSKYVQMHTADRWAENIIT-DLVKTT-------------------------------------------------  635
C. merolae CM3596 TPS                 QNN---DEVKQRKHAADYSYVCEHTTATWAYSFLSDLERASEPVRRLTRLGLGYGVGARLVEF----------------------------  715
G. sulphuraria TPS                    SMT---RHERKLRHDVAYDFVSTHTVKLWSNNFLQ-DLEEVEDNRLS--------------------------------------------  550
G. sulphuraria TPS-1                  QLD---QNEISRKLHADYKYVTAHSTMEWAESFLADLERASEPVNRVTKL-----------------------------------------  665
G. sulphuraria TPS-2                  SMP---RREREIKHWKLYRYV-------WLEVFEF-VFLMIY-------------------------------------------------  630
P. haitanensis TPS1                   SAS---PRERQLKQQKLYRYVTTHTAAFWAQSFVS-ELRELANLE----------------------------------------------  600
P. haitanensis TPS2                   TMS---DAERELKHKKLYRYILMHSSSQWGLNFVS-DLLQYSAARRLAVEKLVRLPVAH--------------------------------  750
P. haitanensis TPS3                   TMP---TMDKRSRDEAAYRFVTTHTAQLWGLNFLE--------------------------------------------------------  525
P. haitanensis TPS4                   QKAIAASPEVELKRRADKAYVSAHSSQQWAQSFL---------------------------------------------------------  650
P. yezoensis TPS-1                    TMP---AMDKRSRDEAAYRFVTTHTAQLWGLNFLE-DLEEIEPVRARMVATPHLDEAALSDSLRSSTKRKLFVLDYDGTLIPFHPLWQLAA  557
P. yezoensis TPS-2                    SAS---PRERQLKQQKLYRYVTTHTAAFWAQSFVS-ELRELANLE----------------------------------------------  600
P. yezoensis TPS-3                    TMS---DAERELKHKKLYRYILMHSSSQWGLNFVS-DLLQYSAA-----------------------------------------------  765
P. yezoensis TPS-4                    QKAIAAAPEVELKRRADKAYVSAHSSQQWAQSFLHDLKEASEPAQVVVKVGPLAGLPG---------------------------------  575
P. umbilicalis TPS-1                  QKALSKAPEVELKRRADKSYVSAHSSQQWAQSFLHDLKEASEPARAVVKVG----------------------------------------  676
P. umbilicalis TPS-2                  TMP---AIDKRSRDEAAYRFVTTHTAQLWGLNFL---------------------------------------------------------  805
C. crispus TPS-1                      EMP---DFERELKHQKLYRHVVMHTASAWGVRFID-DLVKFSAIRKA--------------------------------------------  587
C. crispus TPS-2                      QKKETRAAEIRLKHEADRKYVTLNSSSNWAESFLGDLREASESVKEVVRLGLSR-------------------------------------  600
C. crispus TPS-3                      EMP---AAARATRNDAAYRFVTSHTASLWGFNFLE-DLEQADGAQEAG-------------------------------------------  625
P. tricornutum CCAP1055/1 TPS-1       N---MTEDERIRRIRIASEFVTRVSTQRWALAVMLDLKGVNKNSPVQ--------------------------------------------  786
P. tricornutum CCAP1055/1 TPS-2       S---MGQEEREGRRYRDIDFVSTSPSDKWVKNVLRDLR-----DATLRQQSVGSES-----------------------------------  526
T. pseudonana CCMP1335 TPS-1          T---MSEDERMRRISIASEFVTRVTTQRWALAVMLDLKGVQKNEDAGRYAGA---------------------------------------  490
T. pseudonana CCMP1335 TPS-2          S---MTMNERDARRGRDIDFVSTCPSGLWTRNVLRDLN-----DATHDDSIKMK-------------------------------------  490
T. pseudonana CCMP1335 TPS-3          N---MSKEEREGRHLRDIEFVSSSSSNQWMRNVLRDLRYQCSTDQTDDEKASKQKSS----------------------------------  525
S. japonica TPS                       QKAIAAAPEVELKRRADKAYVSAHSSQQWAQSFLHDLKEASEPAQVVVKVGPLAGLPG---------------------------------  575
S. japonica TPS-2                     QP--MRVELRSERLAQGLEYVTSNTTSYWAAQILTDLKAVGRSADRSILSPVGLGLNFR--------------------------------  676
S. henslowianum TPS                   QKAIAAAPEVELKRRADKAYVSAHSSQQWAQSFLHDLKEASEPAQVVVEVGPLAGLPG---------------------------------  575
U. pinnatifida TPS                    QKAIAAAPEVELKRRADKAYVSAHSSQQWAQSFLHDLKEASEPAQVVVKVGPRAGLPG---------------------------------  575
C. albicans TPS                       TLP---EEKREFNFKKLFTYISKYTSGFWGESFVK-ELYKCNPQKSLRD------------------------------------------  478
C. higginsianum TPS1                  TMS---PEQRETNYKKLERYVFKYTSSWWGQSFVA-EMTRLSQETSQSKTLRNISGNVVGQVSKALHGAIEGASNLTGGKDSADETKQEA-  526
S. cerevisiae TPS                     TLP---DVKKEVNWEKLYKYISKYTSAFWGENFVH-ELYSTSSSSTSSSATKN--------------------------------------  495
F. cylindrus CCMP1102 TPS             S---MDPEEKEGRRYRDLDFVSNSPSDRWVRNVLRDMN-----DAYTASSSGHRRTS----------------------------------  530
V. carteri f. nagariensis TPS-1       KLP---REHRQLRHEKHWRYVSQHTVAYWATSFVAELQRVTKNHVTMKC------------------------------------------  500
C. reinhardtii TPS-1                  SMS---EDERRERHRQNYMHVQTHTAQHWADTFIT-ELNDTHIEADLR-------------------------------------------  545
C. reinhardtii TPS-2                  KLP---REHRALRHDKHWRYVSQHTVAYWATSFVADLQRVTKNHVAMKC------------------------------------------  550
C. reinhardtii TPS-4                  ------------RGERAGRVVYPSPTG----------GGRARGHVA---------------------------------------------  475
O. tauri TPS-1                        TMP---EAEKIERHRSSFSHVSTHTAQAWADTFIS-ELNDTHVEAELRQ------------------------------------------  620
O. tauri TPS-2                        QMS---GHEHEARHEKHWRYVSEHHVGFWAQSCLAELQRVTEKANGNRCYG----------------------------------------  550
A. thaliana TPS-3                     NMP---YEERETRHRVNFKYVKTHSAEKWGFDFLS-ELNDAFDE-----------------------------------------------  460
A. thaliana TPS-4                     NMS---HEEKERKHKINFQYVKTHSTQQWADDFMKLTLTNILCS-----------------------------------------------  475
A. thaliana TPS-5                     IVS---EAEKQMRHEKHHKYVSTHDVAYWARSFIQDLERACGD------------------------------------------------  550
A. thaliana TPS-8                     KMS---ETEKQLRHEKHYHYISTHDVGYWAKSFMQDLERACRDHYSKR-------------------------------------------  550
Z. mays TPS                           TMP---SDEREKRHRHNYAHVTTHTAQDWAETFVF-ELNDTVAE-----------------------------------------------  585
N. tabacum TPS                        NMS---AEEREKRHRHNFLHVTTHTAQEWAETFVS-ELNDTVIEAQQRIRKV---------------------------------------  570
O. sativa TPS                         SMS---EREKQLRHEKHYRYVSTHDVAYWSKSFVQDLERACKDHFRKP-------------------------------------------  575
Z. marina TPS                         SMP---DGEKQLRHGKHYRYVRTHGVSYWSKSFMQDMERTCKDHFKRRCWGI---------------------------------------  565
P. patens TPS                         TMS---DEERKERHRHNFTHVMTHTAQAWAVNFVS-ELNDTYV------------------------------------------------  520
P. patens TPS-X1                      KMS---DEERKERHRHNFTHVTTHTAQAWAHNFVS-ELYDTIVEAELRTLNEPP-------------------------------------  650
C. elegans TPS-1                      TES---KETRTKHGEKINQFLCVHDIDEWSDQFLDPKWTHEVISQCEVKQLGQFYGLMSRTAQVRRQIVECVLKGLPIRPHFRYSLENAKN  920
H. sapiens TPS                        TMT---PEEQRERMRPMREMVRDNNVYRWAGSMLLDAARLRKRGDLDRVTGNGERPSNNNVIPMFERARKAVS------------------  591
F. chinensis TPS                      TMP---KDERELRMKQLRHREREHDVNFWLQSFLKSVDCLADNN-LTPGRLLPLRE-----------------------------------  500
C. floridanus TPS                     TMP---EDERTLRMNHLRRRERIYDVNYWMKSFLQVMGSLEERDSVGA-------------------------------------------  500
B. antarctica TPS                     TMP---VDERTLRMSRLRRREKEHDVDHWMRSFMKAMGSDVEEDDIGTTRLQPVTI-----------------------------------  520
```

Additional Fig. S1 Multiple sequence alignment of members of the TPS family of *P. haitanensis* with proteins associated with other species. The amino acid sequence format was used to compare the site conservation of the TPS domain of different species and the GgpS domain of cyanobacteria. The accession number information corresponding to the protein sequence of different species is shown in Table S1. The different color fonts on the left distinguish different species, black indicates bacteria, light blue indicates cyanobacteria, red indicates red algae, blue indicates diatom, dark yellow indicates brown algae, dark purple indicates fungus, green indicates green algae, deep Green indicates the plant, orange indicates the animal, and the superscript font indicates the functional domain in the corresponding species protein. Based on the crystal structure analysis results of *E. coli* TPS (PDI No. 1GZ5) and *Candida albicans* TPS (PDI No. 5HUT) protein, the sequence above * indicates the binding site associated with the substrate Glucose-6-phosphate, * corresponding column Conserved residues are indicated in green fonts. Above the sequence # indicates the binding site associated with the substrate UDP-glucose, and the conserved residues in the corresponding column of # are indicated in red. The black background white font in the sequence indicates that the conservation between the residues of different species is ≥90%, the gray background white font indicates that the residue is 90%-70%, and the gray background black font indicates that the residue is 70%-50% conservative. The blue background residue indicates the unique site of the cyanobacterial GgpS protein, and the yellow box indicates the red algae, brown algae and diatoms. The residue area of the binding site of the two substrates is relatively low. The pink box indicates the sequence of four TPS members of the algae.
