# Supplementary material for: Putative trehalose biosynthesis proteins function as differential floridoside-6-phosphate synthases to participate in the abiotic stress response in the red alga Pyropia haitanensis
Source: BMC Plant Biol. 2019 Jul 19;19:325. doi: 10.1186/s12870-019-1928-2 (PMC6642608; doi:10.1186/s12870-019-1928-2)
Supplement: Supplementary file 2 — Figure S2. The phylogenetic tree of the only TPS and TPP protein are constructed, respectively. (A) A NJ tree was constructed to show the phylogenetic relationships of the TPS proteins using the functional-related amino acid sequences from prokaryotes, red algae, diatoms, brown algae, fungi, green algae, plants, and animals. (B) A NJ tree was constructed to show the phylogenetic relationships of the TPP proteins using the functional-related amino acid sequences from prokaryotes, red algae, diatoms, brown algae, fungi, green algae, plants, and animals. Their accession numbers are indicated in Additional file 3: Table S1. There were 1,000 bootstrap replicates. The red triangle shows PhTPS1–4. The functional domain in each sequence was retrieved using the Conserved Domain tool in NCBI and is marked by a superscript. (PDF 280 kb) [file 12870_2019_1928_MOESM2_ESM.pdf]

A

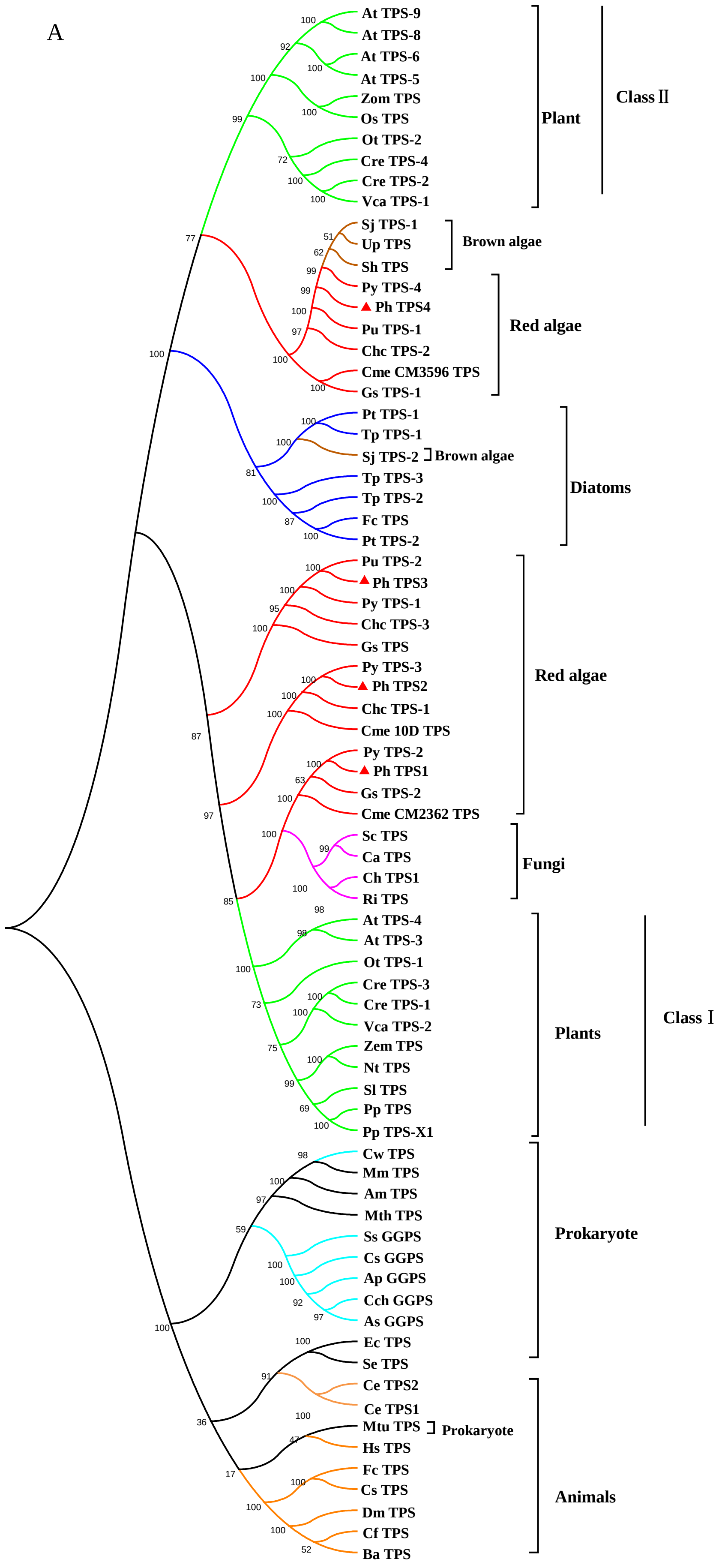
At TPS-9
At TPS-8
At TPS-6
At TPS-5
Zom TPS
Os TPS
Ot TPS-2
Cre TPS-4
Cre TPS-2
Vca TPS-1
Plant
Class Ⅱ
Sj TPS-1
Up TPS
Sh TPS
Brown algae
Py TPS-4
Ph TPS4
Pu TPS-1
Chc TPS-2
Cme CM3596 TPS
Gs TPS-1
Red algae
Pt TPS-1
Tp TPS-1
Sj TPS-2
Brown algae
Tp TPS-3
Tp TPS-2
Fc TPS
Pt TPS-2
Diatoms
Pu TPS-2
Ph TPS3
Py TPS-1
Chc TPS-3
Gs TPS
Py TPS-3
Ph TPS2
Chc TPS-1
Cme 10D TPS
Py TPS-2
Ph TPS1
Gs TPS-2
Cme CM2362 TPS
Red algae
Sc TPS
Ca TPS
Ch TPS1
Ri TPS
Fungi
At TPS-4
At TPS-3
Ot TPS-1
Cre TPS-3
Cre TPS-1
Vca TPS-2
Zem TPS
Nt TPS
Sl TPS
Pp TPS
Pp TPS-X1
Plants
Class Ⅰ
Cw TPS
Mm TPS
Am TPS
Mth TPS
Ss GGPS
Cs GGPS
Ap GGPS
Cch GGPS
As GGPS
Ec TPS
Se TPS
Prokaryote
Ce TPS2
Ce TPS1
Mtu TPS
Prokaryote
Hs TPS
Fc TPS
Cs TPS
Dm TPS
Cf TPS
Ba TPS
Animals

B

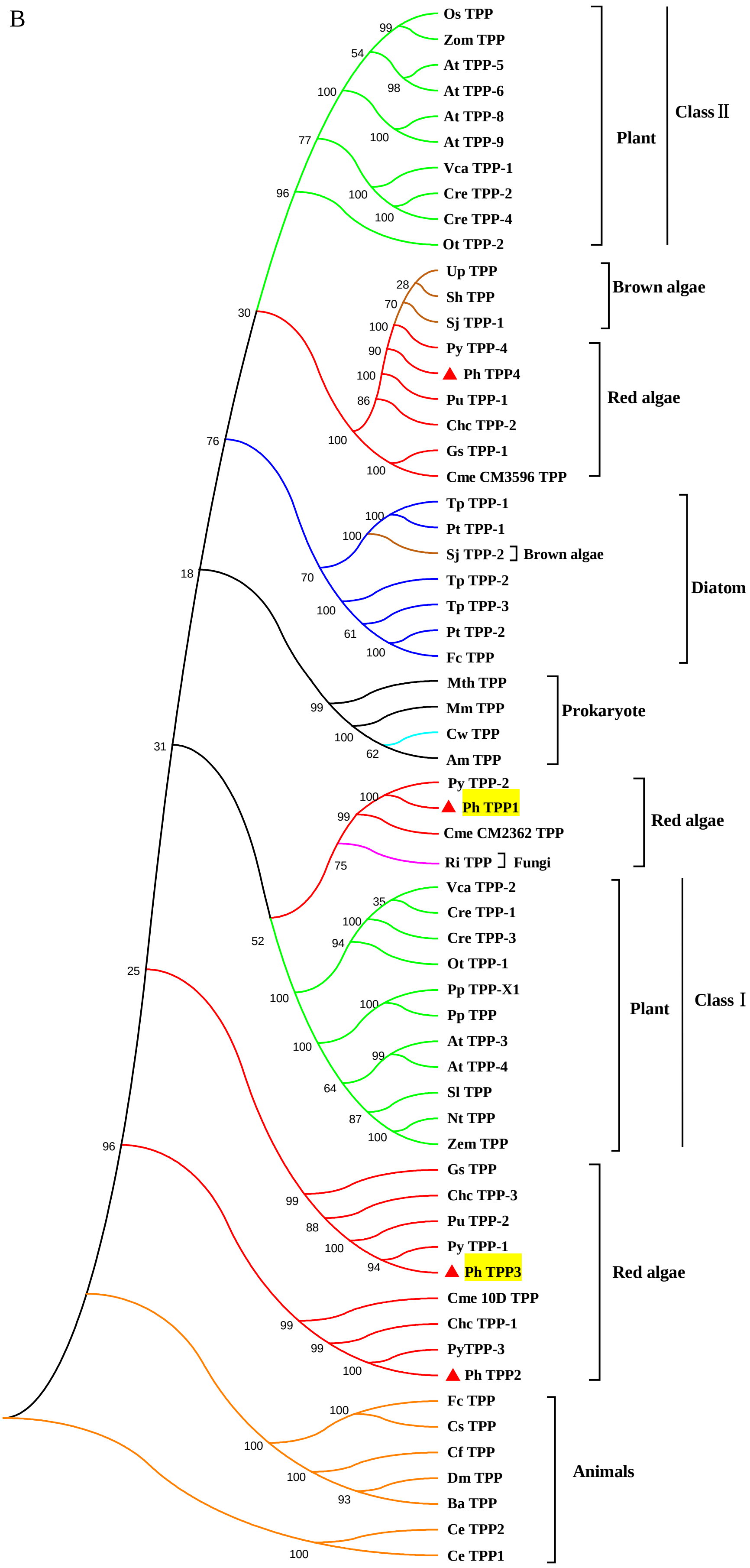

Os TPP
Zom TPP
At TPP-5
At TPP-6
At TPP-8
At TPP-9
Vca TPP-1
Cre TPP-2
Cre TPP-4
Ot TPP-2
Plant
Class Ⅱ
Up TPP
Sh TPP
Sj TPP-1
Brown algae
Py TPP-4
Ph TPP4
Pu TPP-1
Chc TPP-2
Gs TPP-1
Cme CM3596 TPP
Red algae
Tp TPP-1
Pt TPP-1
Sj TPP-2
Brown algae
Tp TPP-2
Tp TPP-3
Pt TPP-2
Fc TPP
Diatom
Mth TPP
Mm TPP
Cw TPP
Am TPP
Prokaryote
Py TPP-2
Ph TPP1
Cme CM2362 TPP
Ri TPP
Fungi
Red algae
Vca TPP-2
Cre TPP-1
Cre TPP-3
Ot TPP-1
Pp TPP-X1
Pp TPP
At TPP-3
At TPP-4
Sl TPP
Nt TPP
Zem TPP
Plant
Class Ⅰ
Gs TPP
Chc TPP-3
Pu TPP-2
Py TPP-1
Ph TPP3
Cme 10D TPP
Chc TPP-1
PyTPP-3
Ph TPP2
Red algae
Fc TPP
Cs TPP
Cf TPP
Dm TPP
Ba TPP
Ce TPP2
Ce TPP1
Animals

Additional Fig. S2 The phylogenetic tree of the only TPS and TPP protein are constructed, respectively. (A) A NJ tree was constructed to show the phylogenetic relationships of the TPS proteins using the functional-related amino acid sequences from prokaryotes, red algae, diatoms, brown algae, fungi, green algae, plants, and animals. (B) A NJ tree was constructed to show the phylogenetic relationships of the TPP proteins using the functional-related amino acid sequences from prokaryotes, red algae, diatoms, brown algae, fungi, green algae, plants, and animals. Their accession numbers are indicated in Table S1. There were 1,000 bootstrap replicates. The red triangle shows PhTPS1–4. The functional domain in each sequence was retrieved using the Conserved Domain tool in NCBI and is marked by a superscript.
