# Supplementary material for: Putative trehalose biosynthesis proteins function as differential floridoside-6-phosphate synthases to participate in the abiotic stress response in the red alga Pyropia haitanensis
Source: BMC Plant Biol. 2019 Jul 19;19:325. doi: 10.1186/s12870-019-1928-2 (PMC6642608; doi:10.1186/s12870-019-1928-2)
Supplement: Supplementary file 3 — Table S1. TPS and GGPS related genes and proteins in different organisms. (PDF 129 kb) [file 12870_2019_1928_MOESM3_ESM.pdf]

Additional Table S1 TPS and GGPS related genes and proteins in different organisms.

Protein Name	Species	Conserved domain	Accession No.
	**Cyanobacteria**		
Cs	*Cyanothece* sp. ATCC51142	GGPS	ACB51740.1
Ap	*Arthrospira platensis*	GGPS	WP_006619700.1
As	*Acaryochloris* sp. CCMEE5410	GGPS	WP_010476679.1
Ss	*Synechococcus* sp. PCC7002	GGPS	ACB00819.1
Cch	*Coleofasciculus chthonoplastes* PCC7420	GGPS	EDX76380.1
Cw	*Crocosphaera watsonii* WH8501	TPS/TPP	EAM50656.1
	**Bacteria**		
Mth	*Methanothrix thermoacetophila* PT	TPS/TPP	ABK14588.1
Am	*Algoriphagus machipongonensis*	TPS/TPP	WP_040303907.1
Mm	*Microscilla marina* ATCC23134 TPS/TPP	TPS/TPP	EAY24641.1
Se	*Salmonella enterica* Virchow str. SL491	TPS	EDZ01753.1
Es	*Escherichia coli* strain K12	TPS	1GZ5 [a]
Mtu	*Mycobacterium tuberculosis*	TPS	P9WN10.1
	**Red algae**		
Cme 10D	*Cyanidioschyzon merolae* strain 10D	TPS/TPP	XP_005537876.1
Cme CM3596	*Cyanidioschyzon merolae* CM3596	CBM20/TPS/TPP	BAM80147.1
Cme CM2362	*Cyanidioschyzon merolae* CM2362	TPS/TPP	BAM81439.1
Gs	*Galdieria sulphuraria*	TPS/TPP	EME29908.1
GS	*Galdieria sulphuraria*	CBM20/TPS-1/TPP-1	EME31717.1
GS	*Galdieria sulphuraria*	TPS-2/TPP-2	EME32832.1
Ccr	*Chondrus crispus*	TPS-1/TPP-1	XP_005712424.1
Ccr	*Chondrus crispus*	TPS-2/TPP-2	CDF40277.1
Ccr	*Chondrus crispus*	TPS-3/TPP-3	CDF33834.1
Py	*Pyropia yezoensis*	TPS1	Contig 2138 [b]
Py	*Pyropia yezoensis*	TPS-2/TPP-2	Contig 4636 [b]
Py	*Pyropia yezoensis*	TPS-3/TPP-3	Contig 27879 [b]
Py	*Pyropia yezoensis*	TPS-4/TPP-4	AAW27916.1
Ph	*Pyropia haitanensis*	TPS1/TPP1	KF147832.1
Ph	*Pyropia haitanensis*	TPS2/TPP2	KM519457.1
Ph	*Pyropia haitanensis*	TPS3/TPP3	KM519458.1
Ph	*Pyropia haitanensis*	CBM20/TPS4/TPP4	KF245464.1
Pu	*Porphyra umbilicalis*	CBM20/TPS-1/TPP-1	OSX79290.1
Pu	*Porphyra umbilicalis*	TPS-2/TPP-2	OSX81421.1
	**Brown alage**		
Sj	*Saccharina japonica*	TPS/TPP	ABG75727.1
Sj	*Saccharina japonica*	CBM20/TPS-2/TPP-2	AGT20052.1
Sh	*Sargassum henslowianum*	TPS/TPP	ADB19856.1
Up	*Undaria pinnatifida*	TPS/TPP	ADB19855.1
	**Diatoms**		
Tp	*Thalassiosira pseudonana* CCMP1335	TPS-1/TPP-1	XP_002288483.1
Tp	*Thalassiosira pseudonana* CCMP1335	TPS-2/TPP-2	XP_002287922.1
Tp	*Thalassiosira pseudonana* CCMP1335	TPS-3/TPP-3	XP_002286273.1
Pt	*Phaeodactylum tricornutum* CCAP1055/1	CBM20/TPS-1/TPP-1	XP_002180425.1
Pt	*Phaeodactylum tricornutum* CCAP1055/1	TPS-2/TPP-2	XP_002183251.1
Fc	*Fragilariopsis cylindrus* CCMP1102	TPS/TPP	OEU17281.1
	**Fungi**		
Sc	*Saccharomyces cerevisiae*	TPS	ABU44492.1
Ca	*Candida albicans*	TPS	5HUT [a]
Ch	*Colletotrichum higginsianum*	TPS	CCF35125.1

Ri	*Rhizophagus irregularis* DAOM197198w	TPS/TPP	EXX70387.1
	**Green alage**		
Vca	*Volvox carteri* f. nagariensis	TPS-1/TPP-1	XP_002945900.1
Vca	*Volvox carteri* f. nagariensis	TPS-2/TPP-2	XP_002953083.1
Cre	*Chlamydomonas reinhardtii*	TPS-1/TPP-1	PNW72009.1
Cre	*Chlamydomonas reinhardtii*	TPS-2/TPP-2	PNW82332.1
Cre	*Chlamydomonas reinhardtii*	TPS-3/TPP-3	XP_001692098.1
Cre	*Chlamydomonas reinhardtii*	TPS-4/TPP-4	XP_001698066.1
Ot	*Ostreococcus tauri*	TPS-1/TPP-1	OUS42733.1
Ot	*Ostreococcus tauri*	TPS-2/TPP-2	OUS46634.1
	**Plants**		
At	*Arabidopsis thaliana*	TPS-3/TPP-3	NP_173143.4
At	*Arabidopsis thaliana*	TPS-4/TPP-4	NP_194485.1
At	*Arabidopsis thaliana*	TPS-5/TPP-5	NP_567538.1
At	*Arabidopsis thaliana*	TPS-6/TPP-6	AAG52003.1
At	*Arabidopsis thaliana*	TPS-8/TPP-8	NP_177186.2
At	*Arabidopsis thaliana*	TPS-9/TPP-9	NP_173799.1
Os	*Oryza sativa* Japonica Group	TPS/TPP	AAT01318.1
Sl	*Selaginella lepidophylla*	TPS/TPP	CAB69548.1
Nt	*Nicotiana tabacum*	TPS/TPP	BAI99252.1
Zem	*Zea mays*	TPS/TPP	AFW82488.1
Pp	*Physcomitrella patens*	TPS-X1/TPP-X1	XP_024377185.1
Pp	*Physcomitrella patens*	TPS/TPP	XP_024375257.1
Zom	*Zostera marina*	TPS/TPP	EU399760.1
	**Animals**		
Hs	*Homo sapiens*	TPS	SJM27984.1
Dm	*Drosophila melanogaster*	TPS/TPP	AAF51020.1
Ce	*Caenorhabditis elegans*	TPS-1/TPP-1	Q7YZT6.1
Ce	*Caenorhabditis elegans*	TPS-2/TPP-2	O45380.3
Cf	*Camponotus floridanus*	TPS/TPP	EFN65057.1
Ba	*Belgica antarctica*	TPS/TPP	JX462664.1
Fc	*Fenneropenaeus chinensis*	TPS/TPP	EU555435.1
Cs	*Callinectes sapidus*	TPS/TPP	ACL00654.1

[a] Accession no. came from PDB database, [b], accession no. came from the *Pyropia yezoensis* genome (http://nrifs.fra.affrc.go.jp/ResearchCenter/5_AG/genomes/nori/).
